# Hybridization-proximity labeling reveals spatially ordered interactions of nuclear RNA compartments

**DOI:** 10.1016/j.molcel.2021.10.009

**Published:** 2022-01-20

**Authors:** Karen Yap, Tek Hong Chung, Eugene V. Makeyev

**Affiliations:** 1 Centre for Developmental Neurobiology, King’s College London, London SE1 1UL, UK

**Keywords:** RNA-containing compartments, proximity biotinylation, ascorbate peroxidase, digoxigenin-binding domain, nucleolus, paraspeckles, perinucleolar compartment, proteome, transcriptome, higher-order nuclear organization

## Abstract

The ability of RNAs to form specific contacts with other macromolecules provides an important mechanism for subcellular compartmentalization. Here we describe a suite of hybridization-proximity (HyPro) labeling technologies for unbiased discovery of proteins (HyPro-MS) and transcripts (HyPro-seq) associated with RNAs of interest in genetically unperturbed cells. As a proof of principle, we show that HyPro-MS and HyPro-seq can identify both known and previously unexplored spatial neighbors of the noncoding RNAs 45S, NEAT1, and PNCTR expressed at markedly different levels. Notably, HyPro-seq uncovers an extensive repertoire of incompletely processed, adenosine-to-inosine-edited transcripts accumulating at the interface between their encoding chromosomal regions and the NEAT1-containing paraspeckle compartment. At least some of these targets require NEAT1 for their optimal expression. Overall, this study provides a versatile toolkit for dissecting RNA interactomes in diverse biomedical contexts and expands our understanding of the functional architecture of the mammalian nucleus.

## Highlights

• HyPro labeling uncovers interactors and spatial neighbors of RNAs of interest
• Protein and RNA partners are identified by mass spectrometry and deep sequencing
• No genetic modifications are required, allowing wider biomedical use
• Interactomes of RNA-containing nuclear bodies are mapped as a proof of principle

## Graphical abstract

**Figure:**
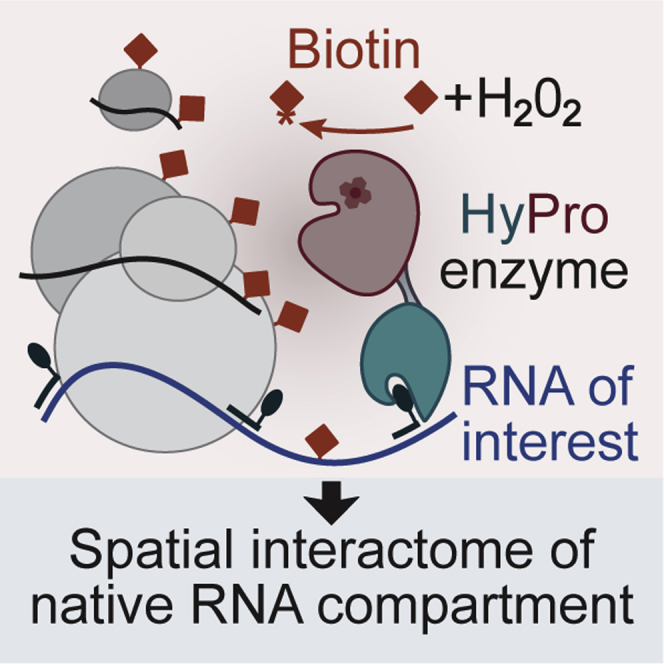

## Teaser

Yap et al. developed a spatial interactome mapping approach relying on recruitment of a compact peroxidase reagent to RNAs of interest and proximity biotinylation in fixed genetically unperturbed cells. The authors show that this technology can identify RNA compartment-specific proteomes and transcriptomes and reveal higher order contacts of transcribed genomic regions.

## Introduction

Subcellular compartmentalization is a fundamental property of life required for optimal efficiency and precise regulation of biological processes (Banani et al., 2017). In addition to classical membrane-bound organelles, eukaryotic cells contain numerous membrane-less assemblies ranging from large multi-subunit complexes to phase-separated condensates. The structure and function of many such compartments depend on RNA molecules (Alberti and Hyman, 2021; Chujo and Hirose, 2017; Mao et al., 2011b; Roden and Gladfelter, 2021; Sawyer et al., 2019; Wilkinson et al., 2020; Yusupova and Yusupov, 2014).

For example, precursors of ribosomal RNAs (pre-rRNAs), including 45S and other processing intermediates, are key constituents of the nucleolus, a nuclear body essential for ribosome biogenesis (Lafontaine et al., 2021; Németh and Grummt, 2018). Another type of nuclear bodies, paraspeckles, assemble around the long noncoding RNA (lncRNA) NEAT1 and participate in the control of gene expression, RNA quality, and microRNA (miRNA) production (Chen and Carmichael, 2009; Fox et al., 2018; Jiang et al., 2017). We have shown that the short tandem repeat-containing RNA PNCTR is critical for structural integrity of the perinucleolar compartment (PNC), which contributes to the regulation of pre-mRNA splicing and cancer cell viability (Yap et al., 2018).

At least some RNA-containing compartments can form specific contacts with various genomic regions, suggesting a role in the higher order nuclear organization (Chen et al., 2018; Maass et al., 2019; Quinodoz et al., 2020; Smith et al., 2020; Takei et al., 2021). The pervasive expression of non-protein-coding sequences in health and disease (Gil and Ulitsky, 2020; Goodwin and Swanson, 2014; Quinn and Chang, 2016; Statello et al., 2021; Uszczynska-Ratajczak et al., 2018; Van Treeck and Parker, 2018) suggests that RNA-dependent compartmentalization may be substantially more widespread and functionally important than currently thought.

Many current techniques for analysis of ribonucleoprotein assemblies rely on isolation of native or crosslinked complexes followed by deep sequencing or mass spectrometry (Cai et al., 2020; Engreitz et al., 2014; Hafner et al., 2021; West et al., 2014). Although ideal for capturing direct binding events, these approaches may be less suitable for discovery of molecular associations on a compartment-wide scale or identifying persistent contacts between different compartments. These tasks can be accomplished by proximity biotinylation of proteins and/or RNAs colocalizing with a “bait” molecule in the cell (Li et al., 2018; Mukherjee et al., 2019; Qin et al., 2021; Ramanathan et al., 2018). For instance, ascorbate peroxidase APEX2 genetically fused with compartment-specific localization signals or RNA interaction domains has been recently used to examine several RNA-containing complexes (Benhalevy et al., 2018; Fazal et al., 2019; Han et al., 2020; Kaewsapsak et al., 2017; Lin et al., 2021; Markmiller et al., 2018; Padrón et al., 2019). However, most proximity-labeling methods rely on expression of recombinant enzymes in living cells, making it difficult to apply this technology to poorly transfectable cell types, non-model organisms, or clinical samples. Moreover, the design and expression levels of the fusion proteins used in these protocols must be carefully optimized to reduce the labeling background, as well as the risk for cytotoxicity and mislocalization artifacts.

### Design

**Figure 1:**
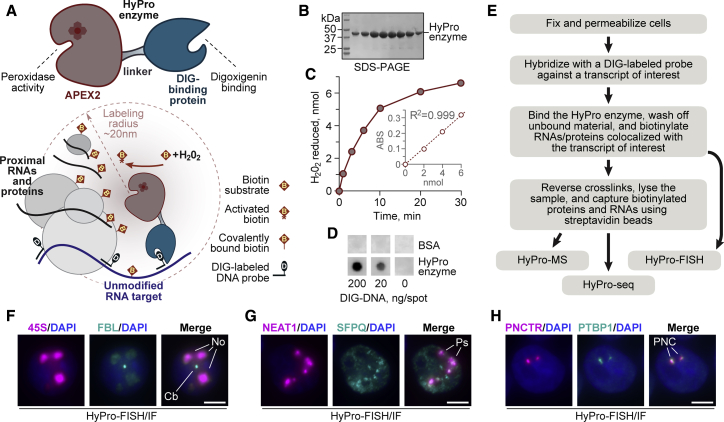
Hybridization-proximity labeling technology for genetically unperturbed samples (A) Top: recombinant HyPro enzyme containing the APEX2 and digoxigenin-binding domains connected by a flexible linker. Bottom: HyPro-labeling approach to identify proteins and RNAs colocalizing with an RNA target of interest. (B) SDS-PAGE analysis of purified HyPro protein peak eluted from a size exclusion chromatography column. (C) Peroxidase activity assay of purified HyPro enzyme. (D) Spot assay showing that HyPro enzyme can bind digoxigenin-labeled DNA while retaining the peroxidase activity. (E) Workflows developed to identify proteins (HyPro-MS) and RNAs (HyPro-seq) colocalized with a transcript of interest and to validate specificity of digoxigenin-labeled probes (HyPro-FISH). (F–H) HyPro-FISH analyses of (F) the 45S pre-rRNA, (G) NEAT1, and (H) the (UC)n-repeated part of PNCTR combined with immunofluorescence detection of nucleolar (No) marker FBL, paraspeckle (Ps) marker SFPQ, and PNC marker PTBP1. As expected, FBL but not 45S is detectable in Cajal bodies (Cb). Image acquisition settings were adjusted according to RNA bait abundance. All images in (F)–(H) are maximum-intensity z stacks. Scale bars, 5 μm. See also Figure S1.

To address these challenges, we developed a straightforward approach for systematic discovery of RNA-protein and RNA-RNA proximity patterns in genetically unmodified samples (Figure 1A). It relies on hybridization of digoxigenin-labeled antisense probes to RNA molecules of interest in chemically fixed cells and binding of a compact custom-designed HyPro enzyme (from hybridization-proximity labeling) to digoxigenin groups. Unbound HyPro is then washed off, and proteins and RNAs physically proximal to the RNA bait are biotinylated *in situ*. Following crosslink reversal, labeled proteins and RNAs are captured on streptavidin beads under denaturing conditions and analyzed using mass spectrometry (HyPro-MS) or RNA sequencing (HyPro-seq). As a proof of principle, we use HyPro-MS and HyPro-seq to profile cellular neighbors of nuclear body-specific lncRNAs expressed at vastly different levels and show that these approaches can uncover new interactions, cellular structures, and gene regulation mechanisms.

## Results

### Development of the HyPro technology

We first devised a bifunctional HyPro protein (Figure 1A) by fusing a bacterial codon-optimized version of APEX2 with the DIG10.3 domain that binds digoxigenin with a subnanomolar affinity (Tinberg et al., 2013). We argued that this compact (<50 kDa) design should provide a better access to crowded molecular environments in fixed samples and a tighter proximity-labeling radius compared with substantially bulkier complexes of antibodies and horseradish peroxidase conjugates currently used for biotinylation *in situ* (Bar et al., 2018; Chen et al., 2018). Recombinant HyPro was expressed in *E. coli* in a soluble form (Figure S1A), and both the APEX2 and the digoxigenin-binding activities of the purified protein were validated *in vitro* (Figures 1B–1D; Figures S1B and S1C).

To develop a fixation/permeabilization procedure that would maintain subcellular organization while allowing efficient extraction of labeled proteins and RNAs, we treated HeLa cells with thiol-cleavable dithio-bis(succinimidyl propionate) (DSP) crosslinking reagent (Xiang et al., 2004) and permeabilized the samples with 70% ethanol (EtOH). The cells were then stained with digoxigenin-labeled oligonucleotide probes against noncoding RNAs 45S, NEAT1 (Table S1), or the (UC)n-repeated part of PNCTR and antibodies against digoxigenin and protein markers of the corresponding nuclear compartments (FBL/nucleolus, SFPQ/paraspeckles, PTBP1/PNC; Figures S1D–S1F). The 45S fluorescence *in situ* hybridization (FISH) signal colocalized with nucleolar FBL, NEAT1 overlapped with paraspeckle-specific SFPQ puncta, and PNCTR and PTBP1 colocalized in the PNC. The same fixation/permeabilization protocol followed by NEAT1/SFPQ staining also revealed characteristic paraspeckle-like structures in a non-transformed epithelial cell line, ARPE-19 (Figure S1G). Combined with the normal appearance of DAPI-stained nuclei, this suggested that DSP/EtOH preserved cellular morphology sufficiently well.

We then investigated whether the HyPro enzyme can be used for proximity biotinylation *in situ* using the HyPro-FISH procedure (Figure 1E). DSP/EtOH-treated HeLa cells were hybridized with the same digoxigenin-labeled 45S, NEAT1, or (UC)n-specific PNCTR probes as above, incubated with HyPro, washed, and briefly exposed to APEX2 substrates, biotin-phenol, and hydrogen peroxide. Subsequent staining of the samples with fluorescently labeled streptavidin and compartment-specific antibodies showed that the biotin groups were deposited at or near the nucleoli, paraspeckles, and PNC, respectively (Figures 1F–1H). HyPro-FISH signal was also localized to the PNC when we used a probe set against non-repetitious PNCTR sequences (PNCTR_NR_; Figure S1H). Conversely, the entire cell was labeled in a control experiment in which DSP/EtOH-treated HeLa cells were incubated without a probe and then infused with HyPro enzyme prior to the biotinylation step (Figure S1I).

As a further control for HyPro-FISH specificity, we scrambled the 45S-specific oligonucleotide sequences using two online programs (https://www.genscript.com/tools/create-scrambled-sequence and https://www.invivogen.com/sirnawizard/scrambled.php; Table S1) and labeled the resultant Scrambled1 and Scrambled2 sets with digoxigenin. Both scrambled controls produced no detectable HyPro-FISH staining in HeLa cells, while the original 45S set highlighted the nucleoli, as expected (Figure S1J). Similar difference between the 45S-specific and scrambled sets was observed when we repeated the experiment in human induced pluripotent stem cells (hiPSCs; Figure S1K). HyPro-FISH analysis of ARPE-19 cells with the NEAT1-specific set generated a paraspeckle-like pattern with no signal detected in the no-probe control (Figure S1L). Of note, HyPro-FISH also gave rise to nuclear body-specific labeling in samples fixed with formaldehyde and permeabilized with Triton X-100 (Figures S1M–S1O).

These results indicate that the HyPro technology can specifically label RNA-containing compartments in a variety of biological samples.

### HyPro-MS identifies compartment-specific proteomes

To assess the utility of the newly developed procedure for compartment-specific proteomics, we HyPro-labeled HeLa cells with probes against either the 45S or NEAT1 RNAs estimated to be expressed at >10^4^ and ∼10^3^ copies per cell, respectively (Chujo et al., 2017; Jackson et al., 2000). We then reversed the DSP crosslinks by dithiothreitol (DTT) and analyzed biotinylated proteins using immunoblotting with a streptavidin detection reagent (Figure S1P). The 45S and NEAT1 samples produced distinctive labeling patterns that differed from each other and also from the HyPro-infused control prepared as in Figure S1I. No signal was detected in samples incubated without a probe or when either of the two substrates was omitted from the labeling reaction. Similarly, both Scrambled1 and Scrambled2 variants of the 45S probe set produced no detectable biotinylation in both HeLa and hiPSC samples (Figure S1Q).

**Figure 2:**
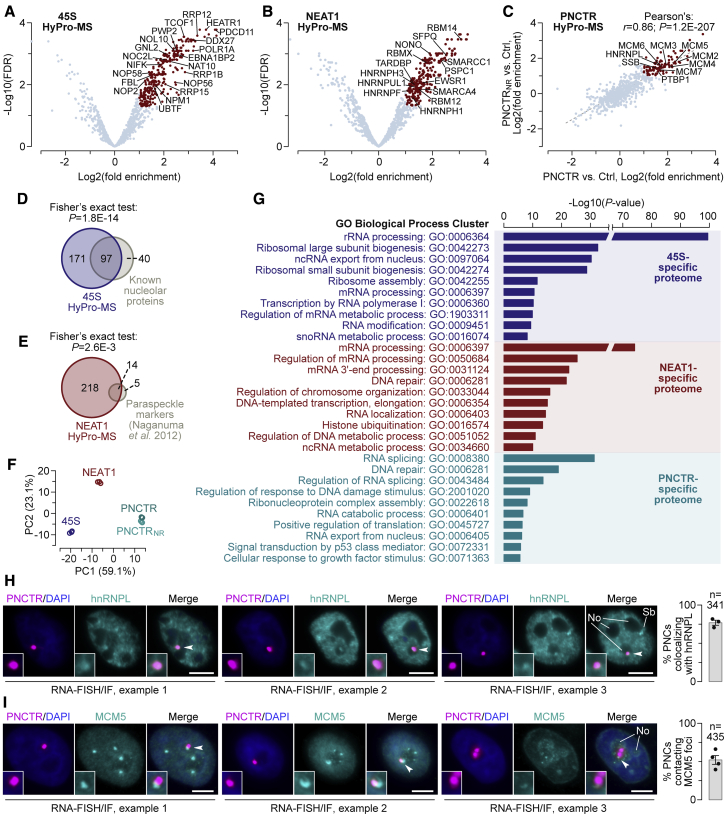
HyPro-MS identifies both known and new RNA-proximal proteins (A and B) Volcano plots showing that HyPro-MS with (A) 45S-specific and (B) NEAT1-specific probes detects known nucleolar and paraspeckle proteins, respectively. (C) Scatterplot showing significant correlation between HyPro-MS experiments carried out with probes against either (UC)n repeats (PNCTR; x axis) or a non-repetitious (NR) part of PNCTR (PNCTR_NR_; y axis). Examples of markers expected to localize to the PNC (PTBP1 and SSB) and novel PNCTR-proximal components (hnRNPL and MCM proteins) are labeled in the top right quadrant. In (A)–(C), red dots denote proteins enriched in lncRNA-specific labeling experiments compared with the HyPro infusion control (>2-fold, FDR < 0.05), and gray dots denote the rest of the proteins detected by mass spectrometry. (D and E) Venn diagrams showing that (D) nucleolar (https://www.proteinatlas.org) and (E) classical paraspeckle markers (Naganuma et al., 2012) are significantly over-represented among the 45S- and NEAT1-labeled proteins (>2-fold, FDR < 0.05), respectively. (F) Principal-component analysis of control-normalized log_2_-transformed protein abundance values for 45S, NEAT1, and PNCTR (UC)n repeat-specific (PNCTR) and non-repetitious (PNCTR_NR_) HyPro-MS experiments. Note tight clustering of the data according to the RNA bait identity. (G) Top 10 Metascape Gene Ontology clusters for 45S-, NEAT1-, and PNCTR1-specific proteins from Table S2. (H and I) Combined RNA-FISH/immunofluorescence analyses showing that (H) PNCTR lncRNA colocalizes with hnRNPL protein and (I) PNCTR-labeled compartments often contact large MCM5-containing structures. Three representative examples are shown for each staining and the data are quantified for three or four staining experiments (341–431 cells in total). No, nucleolus. Scale bars, 5 μm. The arrowheads indicate parts of the images magnified 2-fold in the insets. The main images in (H) and the close-ups in (H) and (I) show individual optical planes. The main images in (I) are maximum-intensity z stacks. Quantifications on the right in (H) and (I) are shown as means ± SD. See also Figures S2 and S3 and Table S2.

We then repeated the experiment in HeLa cells, captured biotinylated proteins on streptavidin beads, and analyzed the 45S- or NEAT1-proximal proteomes in triplicate by label-free mass spectrometry (HyPro-MS; Figure 1E). Comparison of the compartment-specific data with the HyPro infusion control identified 285 and 232 proteins enriched in the 45S and NEAT1-labled samples >2-fold with false discovery rate (FDR) < 0.05. Notably, the two proteomes contained numerous nucleolar or paraspeckle markers, respectively, including FBL and SFPQ (Figures 2A and 2B).

Encouraged by the 45S and NEAT1 data, we turned to PNCTR, a substantially less abundant lncRNA typically present in <50 copies per cell (Yap et al., 2018). HyPro-MS analysis using the (UC)n repeat-specific PNCTR probe revealed 138 proteins enriched versus the HyPro infusion control (>2-fold, FDR < 0.05). The PNCTR_NR_ probe set produced 129 PNCTR-enriched proteins (>2-fold, FDR < 0.05). Of these, 100 proteins were also enriched in the (UC)n-specific HyPro-MS, with a correlation coefficient between the two experiments of *r* = 0.86 (p = 1.2E-207) (Figure 2C). Both enriched sets included the classical PNC marker PTBP1 and the SSB/La protein interacting with Pol III transcripts known to accumulate in the PNC (Matera et al., 1995; Pollock and Huang, 2010; Yap et al., 2018) (Figure 2C).

Consistent with the nuclear localization of 45S, NEAT1, and PNCTR, nuclear proteins were over-represented in all RNA-proximal proteomes (Figure S2A). However, the composition of the enriched sets depended on the bait identity. Known nucleolar proteins were enriched in the 45S (Figure 2D; p = 1.8E-14, Fisher’s exact test), but not NEAT1 (p = 0.065) or PNCTR proteomes (p = 0.82 for intersected [UC]n and non-repetitious datasets). Paraspeckle markers were enriched in the NEAT1 (Figure 2E; p = 2.6E-3) but not 45S (p = 0.35) or PNCTR (p = 0.072) proteomes. The NEAT1 HyPro-MS proteome was also enriched for proteins previously identified in NEAT1-specific CHART-MS experiments (West et al., 2014) (Figure S2B). Moreover, paraspeckle and nucleolar markers partitioned to correct quartiles in a side-by-side volcano plot analysis of the NEAT1 and 45S HyPro-MS data (Figure S2C). Most nucleolar proteins and the core paraspeckle components SFPQ, NONO, RBM14, and PSPC1 survived the significance testing in this comparison with the >1.5-fold and FDR < 0.1 cutoffs (Figure S2C). Consistent with compartment-specific labeling, specificity indices (enrichment in a given compartment divided by the sum of enrichment values in all three compartments; see STAR Methods) for HyPro-labeled proteins were significantly higher compared with unlabeled proteomes (Figure S2D). Finally, principal-component analysis showed tight clustering of the HyPro-labeled samples according to the RNA bait identity (Figure 2F).

Further analysis of high-confidence protein sets with specificity indices for a given compartment exceeding those for other compartments (Table S2) showed enrichment of nucleolus-specific Gene Ontology terms for 45S and various nucleic acid metabolism-related terms for NEAT1 and PNCTR (Figure 2G). Molecular Complex Detection (MCODE) analysis (Zhou et al., 2019b) identified several protein interaction modules containing known nucleolar markers in the case of 45S and paraspeckle markers in the case of NEAT1 (Figures S2E–S2G).

PNCTR-specific MCODE detected a large PTBP1 module and, surprisingly, two modules comprising the entire MCM DNA helicase complex and its interaction partners (Figure S2G). We selected one example from each module, hnRNPL, MCM5, and MCM2, for validation. hnRNPL was detectable throughout the nucleoplasm and in several nuclear foci (Figure 2H). Notably, prominent hnRNPL foci colocalized with PNCTR-stained PNCs. hnRNPL also accumulated in distinct sites possibly corresponding to the SAM68 nuclear bodies (Rajan et al., 2009). MCM5 and MCM2 localized to the nucleus with varying intensity of detergent-resistant nucleoplasmic staining (Figure S3), likely reflecting the cell cycle-dependent MCM dynamics (Prasanth et al., 2004). In cells with relatively low nucleoplasmic levels of these proteins—expected for the S and the G2 phases—MCM5 and MCM2 tended to cluster in the perinucleolar space, often forming discrete foci (Figure 2I; Figure S3). Notably, when such foci occurred near a nucleolus “decorated” with a PNC, the two compartments tended to contact each other (Figure 2I; Figure S3).

We concluded that HyPro-MS allows discovery of both compartment-localized and compartment-proximal proteins.

### Characterization of compartment-specific transcriptomes by HyPro-seq

**Figure 3:**
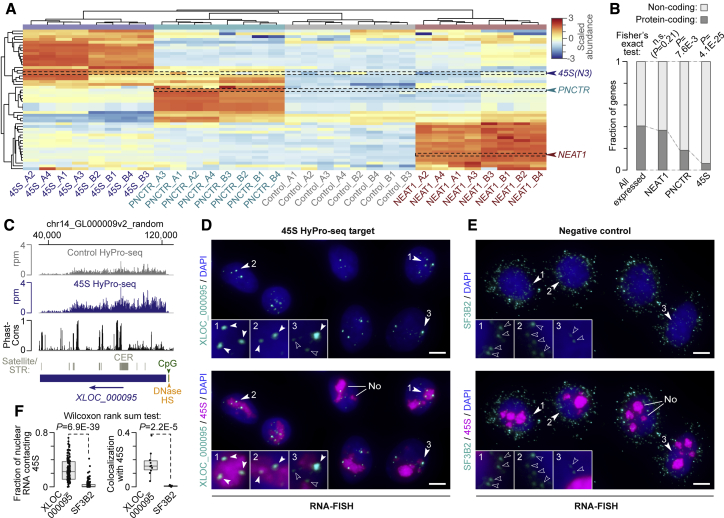
HyPro-seq identifies characteristic repertoires of RNA bait-proximal transcripts (A) Heatmap showing that 45S, PNCTR, and NEAT1 HyPro-seq labels distinctive sets of transcripts including the RNA “baits” themselves. Also note consistent clustering of technical (1–4) and biological (A and B) replicates. (B) 45S and PNCTR, but not NEAT1, tend to associate with non-coding transcripts. (C) Transcribed *XLOC_000095* locus identified by HyPro-seq as a 45S proximity partner. Note stronger RPM-normalized signal in the 45S track compared with the control (∼1.9-fold enrichment, FDR = 6.49E-255). The PhastCons track shows *XLOC_000095* sequences conserved across vertebrates, and the Satellite/STR track indicates the position of the ∼4.7 kb CER satellite unit. The green and orange arrowheads mark a CpG island and a DNase hypersensitive cluster (ENCODE HeLa-S3 data; http://genome.ucsc.edu) upstream of the *XLOC_000095* transcription start. (D and E) RNA-FISH analyses of HeLa cells suggesting that (D) XLOC_000095 but not (E) control SF3B2 transcripts are associated with nucleoli (No) labeled with 45S-specific probes. Scale bars, 5 μm. The arrowheads in the main images indicate areas magnified 3-fold in the insets. Open arrowheads in the insets mark diffraction-limited signals likely corresponding to individual RNA molecules; filled arrowheads, larger foci likely containing several XLOC_000095 molecules. Main images are z stacks; insets, individual optical sections. (F) Quantification of the data in (D) and (E). Left: fractions of nuclear XLOC_000095 or SF3B2 signals forming microscopic contacts with 45S-stained nucleoli (n ≥ 137 DAPI-stained nuclei per sample). Right: weighted Manders’ coefficients comparing colocalization of total XLOC_000095 or SF3B2 signals with 45S for n ≥ 9 randomly selected fields per sample. Data were compared using a two-tailed Wilcoxon test. See also Figure S4 and Table S3.

To find out if the HyPro technology can identify compartment-associated RNAs, we analyzed HeLa 45S, NEAT1, and PNCTR HyPro-labeled transcripts using high-throughput sequencing (HyPro-seq; Figure 1E). Heatmap analysis showed tight clustering of replicated HyPro-seq experiments according to the bait identity (Figure 3A). Each compartment contained a distinctive set of labeled transcripts, including the RNA bait itself (Figure 3A). Bait enrichment was also evident in read per million (RPM) normalized HyPro-seq coverage data (Figure S4A).

Although our RNA extraction protocol was optimized for longer RNAs, some small nucleolar RNAs (snoRNAs) involved in rRNA metabolism (Bouchard-Bourelle et al., 2020) were also enriched in the 45S samples and PNC-associated Pol III transcripts (Matera et al., 1995; Pollock and Huang, 2010), in the PNCTR samples (Figure S4B). Consistent with the role of paraspeckles in miRNA biogenesis (Jiang et al., 2017), we also detected somewhat increased coverage of precursor sequences bordering abundant miRNAs in the NEAT1 samples (Figure S4B).

We benchmarked HyPro-seq by comparing 45S-labeled transcripts with publicly available APEX-seq data from HEK293T cells expressing nucleolus-localized APEX2 (Fazal et al., 2019). Despite the cell line difference, significantly labeled APEX-seq targets (>1.5-fold enrichment, FDR < 0.05) were over-represented among 45S HyPro-seq transcripts enriched >1.5-fold compared with the HyPro infusion control (Figure S4C). The overlap was significant for a wide range of 45S HyPro-seq FDR cutoffs (0.1 to 1E-14), peaking at 1E-10 (Figure S4C). We therefore used the >1.5-fold enrichment and FDR < 1E-10 HyPro-seq cutoffs in our subsequent analyses.

Applying these stringent cutoffs to the 45S, NEAT1, and PNCTR datasets identified 178, 267, and 33 proximity targets, respectively (Table S3). Notably, noncoding transcripts were significantly over-represented among the 45S and PNCTR HyPro-seq targets, whereas NEAT1 labeled protein-coding and noncoding transcripts with comparable efficiencies (Figure 3B). Consistent with the spatial proximity between the nucleolus and the PNC, the 45S-labeled transcripts included PNCTR and, vice versa, 45S was a top PNCTR target (Table S3).

Several 45S-proximal lncRNAs contained CER-family satellite repeats along with conserved non-repetitious sequences (Table S3). CER repeats are enriched on the p-arms of all five acrocentric chromosomes encoding 45S RNA; however, possible biological functions of these sequences remain poorly understood (Floutsakou et al., 2013). We selected an abundant member of this family, lncRNA XLOC_000095, for experimental validation (Figure 3C). Dual-color RNA-FISH and RNA-FISH/immunofluorescence analyses showed that the XLOC_000095 transcripts concentrated in bright foci forming extensive nucleolar contacts (Figures 3D–3F; Figure S4D). As their number per nucleus often exceeded 3, these perinucleolar structures were likely distinct from the PNC, which is typically present in one to three copies per HeLa cell (Yap et al., 2018). Nucleolar proximity of XLOC_000095 was not a staining artifact, as a control probe set against an abundant protein-coding transcript, SF3B2, produced nuclear and cytoplasmic single-molecule signals that seldom contacted the nucleolus (Figures 3D–3F).

These data suggest that HyPro-seq is a useful discovery tool for compartment-specific transcriptomics.

### HyPro-seq uncovers extensive genomic clustering of RNA compartment-proximal targets

**Figure 4:**
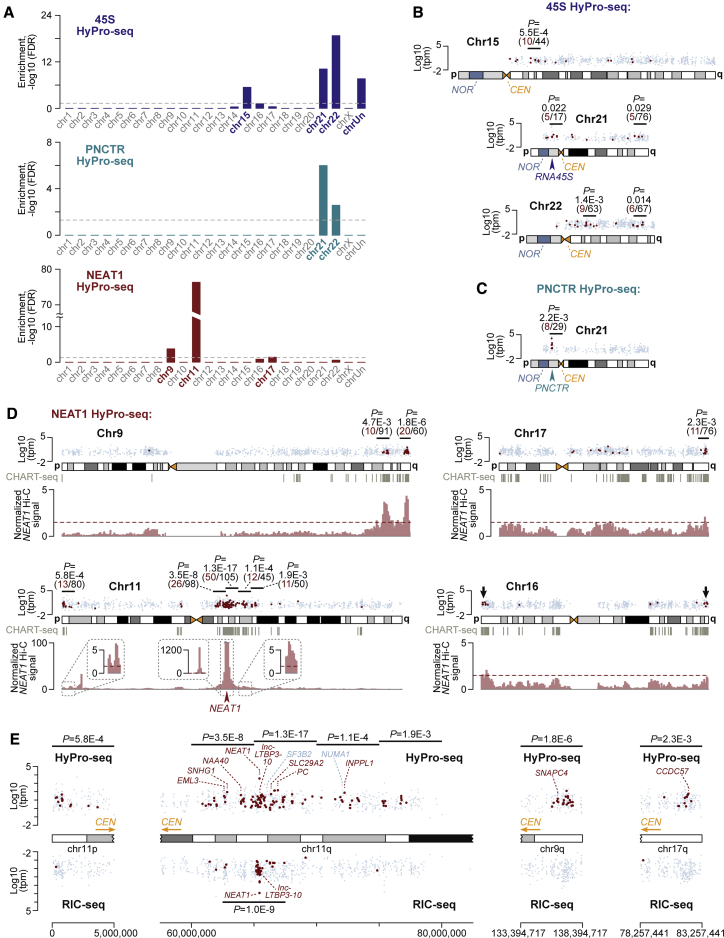
Extensive genomic clustering of proximity-labeled transcripts (A) Top: 45S-proximal transcripts are over-represented on acrocentric chr15, chr21, and chr22 encoding repeated *45S* sequences in the nucleolus organizer regions (NORs). At least some of the unassigned contigs (chrUn) also showing significant enrichment may originate from acrocentric chromosome sequences missing from the current genome assembly (Table S3; Floutsakou et al., 2013). Middle: PNCTR HyPro-seq labeled transcripts are over-represented on chromosomes 21 and 22, both encoding *PNCTR*-like rRNA intergenic sequences. Bottom: NEAT1 HyPro-seq hits are significantly enriched on chr9, chr11, and chr17. In all three panels, significance was analyzed using one-sided Fisher’s exact test, and p values were corrected for multiple testing using the FDR method. (B–D) Chromosomal clustering of HyPro-seq targets in HeLa cells. Shown are log_10_-transformed transcript per million (TPM) values of all detectably expressed genes (gray dots) and HyPro-seq labeled transcripts (red dots; >1.5-fold up, FDR < 1E-10). Black horizontal lines mark non-overlapping 5 Mb sliding windows containing significantly larger than expected numbers of HyPro-seq hits. We identified these regions by sliding a 5 Mb window with 2.5 Mb step and performing one-sided Fisher’s exact test, with p values and numbers of labeled (red) and all expressed genes (black) shown on the top. (D) also shows DNA sequences interacting with NEAT1 RNA according to a CHART-seq study (gray vertical lines; West et al., 2014) and crosslinking with the *NEAT1* locus in a Hi-C dataset (light-red histograms; Rao et al., 2014). The histograms are normalized to the median Hi-C signal for chr11 to reveal genomic regions crosslinking with *NEAT1* with a relatively high efficiency. Peaks exceeding the 1.5 × chr11 median cutoff (dashed red line) often match clustered NEAT1 HyPro-seq hits. CEN, centromeres. Arrowheads, *45S*, *PNCTR*, and *NEAT1* loci. Black arrows, chr16 telomere-proximal genes encoding NEAT1-labeled transcripts. (E) Unlike NEAT1 HyPro-seq, which labels genetically distant clusters of genes, high-quality RIC-seq hits are concentrated in a narrow chr11q region immediately adjacent to the *NEAT1* locus, the only part of the genome significantly enriched in the sliding window analysis introduced above. Of the ten NEAT1 HyPro-seq genes marked in this panel and used in the subsequent validation experiments, only two are short-listed by RIC-seq with comparable stringency: NEAT1 itself and its immediate genetic neighbor, lnc-LTBP3-10. Also shown are two control genes, *SF3B2* and *NUMA1*, unlabeled by HyPro despite their relative genetic proximity to the *NEAT1* locus. Black horizontal lines, non-overlapping 5 Mb sliding windows containing significantly larger than expected numbers of HyPro-seq or RIC-seq hits. Red, detectably expressed genes passing significance cutoffs; light blue, the rest of detectably expressed genes. See also Figure S5 and Tables S3 and S4.

Strikingly, genes encoding the three sets of HyPro-labeled transcripts were distributed in the genome in a highly non-uniform manner. This included gene enrichment in a subset of chromosomes (Figure 4A) and clustering in specific chromosomal regions identified using a sliding window approach (Figures 4B–4D). The 45S targets formed one or two statistically significant clusters on chr15, chr21, and chr22 (i.e., chromosomes known to encode the *45S pre-rRNA* genes themselves) (Figure 4B). Many PNCTR-labeled transcripts originated from chr21 or chr22, forming a tight cluster around a PNCTR-encoding *45S pre-rRNA* intergenic spacer on the p-arm of chr21 (Figure 4C).

Significant clusters of NEAT1 target genes mapped to a ∼16 Mb region on the q-arm of chr11 comprising the *NEAT1* locus itself and telomere-proximal parts of chr11p, chr9q, and chr17q (Figure 4D). The tendency of NEAT1-labeled transcripts to be encoded near telomeres was also apparent for several other chromosomes, including chr16 (Figure 4D). Although these additional clusters failed to reach statistical significance individually, enrichment of the NEAT1 but not the 45S or PNCTR targets near telomeres was readily detectable in a genome-wide analysis (Figure S5A).

To assess the probability of discovering gene clusters by chance, we compared median intergenic distances for real HyPro-seq hits with distributions obtained by repeated random sampling (n = 10,000) of the same number of genes per chromosome from all detectably expressed genes. In all three cases, the simulated distributions were shifted toward larger values compared with the real medians, with the largest distance between simulated and real medians (Δmed) observed for NEAT1 (Figure S5B).

The presence of clusters suggested that HyPro-seq might label transcripts produced locally from genomic regions spatially proximal to corresponding nuclear bodies in the interphase nucleus. Indeed, genes encoding 45S-labeled transcripts were significantly enriched in nucleolus-associated chromosomal domains (NADs; Németh et al., 2010; Figure S5C). Similarly, NEAT1 HyPro-seq targets often overlapped DNA regions crosslinking to the NEAT1 RNA in a published CHART-seq study (Figure 4D; Figure S5D). As paraspeckles assemble in close vicinity of the *NEAT1* gene (Clemson et al., 2009; Mao et al., 2011a), we examined chromatin interactions of the *NEAT1* locus in genome-wide chromosome conformation capture (Hi-C) data (Rao et al., 2014). Strikingly, clustered NEAT1 HyPro-seq genes residing on chr11 and elsewhere in the genome crosslinked to the *NEAT1*-containing chr11q region with a significantly higher efficiency compared with all detectably expressed genes (Figure 4D; Figure S5E).

Two mechanisms may account for clustering of NEAT1 HyPro-seq genes: (1) Physically proximal transcripts may interact with the NEAT1 RNA directly, possibly before it is sequestered in paraspeckles. (2) Alternatively, target transcripts may localize near paraspeckles without forming molecular contacts with NEAT1. To distinguish between these possibilities, we compared our NEAT1 data with transcripts crosslinked to NEAT1 in a recent RIC-seq study (Cai et al., 2020). As a group, NEAT1 HyPro-seq genes showed a significantly higher incidence of NEAT1-specific crosslinks (Figure S5F). This was evident for both expression-normalized numbers and densities of NEAT1-hybrid RIC-seq reads.

Further analysis of the top 267 RIC-seq hits with the highest NEAT1-hybrid read density (i.e., the same number as in our NEAT1 HyPro-seq list) showed that these NEAT1 interactors tended to cluster in a narrow band around the *NEAT1* locus rather than remote genomic locations (Figure 4E; Figure S5G). Simulation of intergenic distances suggested that although the top-scoring NEAT1 RIC-seq genes clustered tighter than expected by chance, their Δmed value was noticeably smaller compared with NEAT1 HyPro-seq (14.0 versus 31.2 Mb; Figure S5H). A similar result was obtained when we simulated intergenic distances for NEAT1 CHART-seq data (Δmed = 11.7 Mb; Figure S5I).

Thus, HyPro-seq may reveal spatially ordered proximity patterns undetectable by crosslinking-based approaches.

### NEAT1 HyPro-labeled transcripts are retained near paraspeckles

To illuminate the mechanisms underlying spatial association between the NEAT1 compartment and its proximity-labeled targets, we analyzed ten NEAT1 HyPro-seq candidates—mRNAs NAA40, CCDC57, PC, SLC29A2, EML3, INPPL1, and SNAPC4; lncRNAs lnc-LTBP3-10 and SNHG1; and NEAT1 RNA itself (Table S4)—using dual-color RNA-FISH. Of these, only NEAT1 and its immediate genetic neighbor lnc-LTBP3-10 were short-listed by RIC-seq with a high stringency (Figure 4E). RIC-seq crosslinking efficiency of the remaining eight targets was comparable with that of NEAT1 HyPro-unlabeled controls SF3B2, NUMA1, and XLOC_000095 (Table S4).

**Figure 5:**
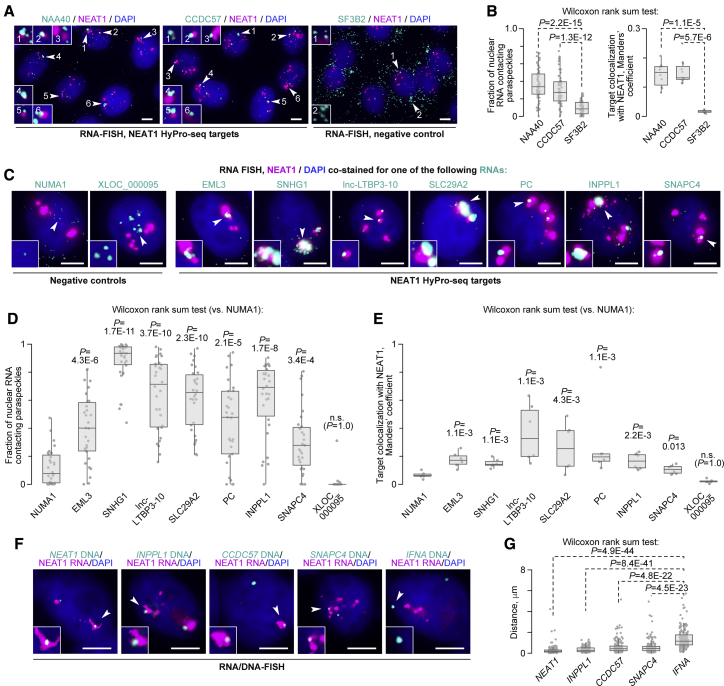
NEAT1 HyPro-seq targets tend to localize near paraspeckles (A) RNA-FISH analyses of HeLa cells for NEAT1 and NEAT1 HyPro-seq targets NAA40 (chr11q) and CCDC57 (chr17) or a chr11q-encoded negative control, SF3B2. Note that NAA40 and CCDC57 often cluster near NEAT1-positive paraspeckles, and SF3B2 tends to be scattered throughout the nucleus and the cytoplasm as diffraction-limited single-molecule spots. Insets: 4× close-ups of the areas marked by the arrowheads in the main image. Main images are maximum-intensity z stacks; close-ups are single optical sections. (B) Quantification of the data in (A) showing that NAA40 and CCDC57 colocalize with NEAT1 significantly better than SF3B2. Left: fractions of nuclear RNA signals forming microscopic contacts with NEAT1-stained paraspeckles (n ≥ 60 DAPI-stained nuclei per sample). Right: weighted Manders’ coefficients comparing colocalization of RNA targets with NEAT1 for n ≥ 10 randomly selected fields per sample. Data were compared using a two-tailed Wilcoxon test. (C) RNA-FISH analyses showing that, similar to NAA40 and CCDC57, NEAT1 HyPro-labeled transcripts EML3, SNHG1, lncLTBP3, PC, SLC29A2, and INPPL1 (all from chr11q) and SNAPC4 (chr9) tend to cluster near paraspeckles. Conversely, the negative control NUMA1 encoded on chr11q near *INPPL1* (see Figure 4E) is scattered in the form of single RNA molecules. As expected, the perinucleolar lncRNA XLOC_000095 identified by 45S HyPro-seq aggregates in a distinct part of the nucleus. Insets: 2× close-ups of the marked areas. Main images are maximum intensity z stacks; close-ups are single optical sections. (D) Fractions of nuclear RNA signals in (C) forming microscopic contacts with NEAT1-positive paraspeckles (n ≥ 29 DAPI-stained nuclei per sample). (E) Weighted Manders’ coefficients for RNA target colocalization with NEAT1 in (C) calculated for n = 6 randomly selected fields per experiment. Data in (D) and (E) are compared with the NUMA1 control using a one-tailed Wilcoxon test. (F) FISH staining of HeLa cells for NEAT1 RNA and DNA loci encoding NEAT1 itself or its genetically distant HyPro-seq targets INPPL1 (chr11q, ∼6.8 Mb away from *NEAT1*), CCDC57 (chr17q), and SNAPC4 (chr9q). As a negative control, we used chr9p-encoded *IFNA* gene cluster not predicted to contact paraspeckles. Note that at least some alleles of the *INPPL1*, *CCDC57*, and *SNAPC4* genes, but not *IFNA*, are physically proximal to paraspeckles. Scale bars in (A), (C), and (F), 5 μm. Insets: 2× close-ups of the marked areas. Both the main images and the close-ups are single optical sections. (G) Two-tailed Wilcoxon test comparison of the nearest cellular distances between DNA loci and NEAT1-positive paraspeckles in (F). The data are obtained from n ≥ 152 nuclei per sample and plotted with the medial distance increasing from left to right. Although not reaching the degree of proximity observed for *NEAT1*, all three NEAT1 HyPro-seq loci are significantly closer to paraspeckles than *IFNA*. See also Table S4.

We first co-stained HeLa cells with RNA-FISH probe sets against NEAT1 and the NAA40 or CCDC57 targets from the chr11q and chr17q, respectively, or the SF3B2 control encoded on chr11q relatively close to the *NEAT1* gene (Figure 4E). The NAA40 and CCDC57 probes gave rise to diffraction-limited single-molecule spots and noticeably larger RNA foci often found near NEAT1-positive paraspeckles and likely containing multiple (pre-)mRNA transcripts (Figure 5A). On the other hand, SF3B2 was mainly scattered throughout the cell as single molecules (Figure 5A; see also Figure 3E). Quantitation of the RNA-FISH data showed that a significantly larger fraction of NAA40 and CCDC57 RNAs contacted NEAT1-stained paraspeckles compared with SF3B2 (Figure 5B).

Similar results were obtained when we extended RNA-FISH analyses to other NEAT1 proximity-labeled candidates or used different negative controls (Figure 5C). All NEAT1 HyPro-seq transcripts formed bright RNA foci often residing near paraspeckles. The negative control NUMA1 was similar to SF3B2 in that it was detected predominantly as randomly distributed individual molecules. As expected, the XLOC_000095 foci occurred at a considerable distance from paraspeckles. These observations were confirmed by appropriate statistical analyses (Figures 5D and 5E).

Collectively, these analyses suggest that NEAT1 HyPro-seq identifies a specific set of transcripts retained in paraspeckle vicinity.

### NEAT1 HyPro-labeled transcripts are often poorly processed and A-to-I edited

Newly synthesized RNAs can accumulate near the transcription site, especially in the case of their incomplete processing (Bhatt et al., 2012; Custódio et al., 1999). The CHART-seq and Hi-C data (Figure 4D; Figures S5D and S5E), as well as our RNA/DNA-FISH analyses showing that genes encoding NEAT1 HyPro-seq targets often reside near paraspeckles (Figures 5F and 5G), were consistent with transcription site-proximal retention of NEAT1-labeled transcripts.

**Figure 6:**
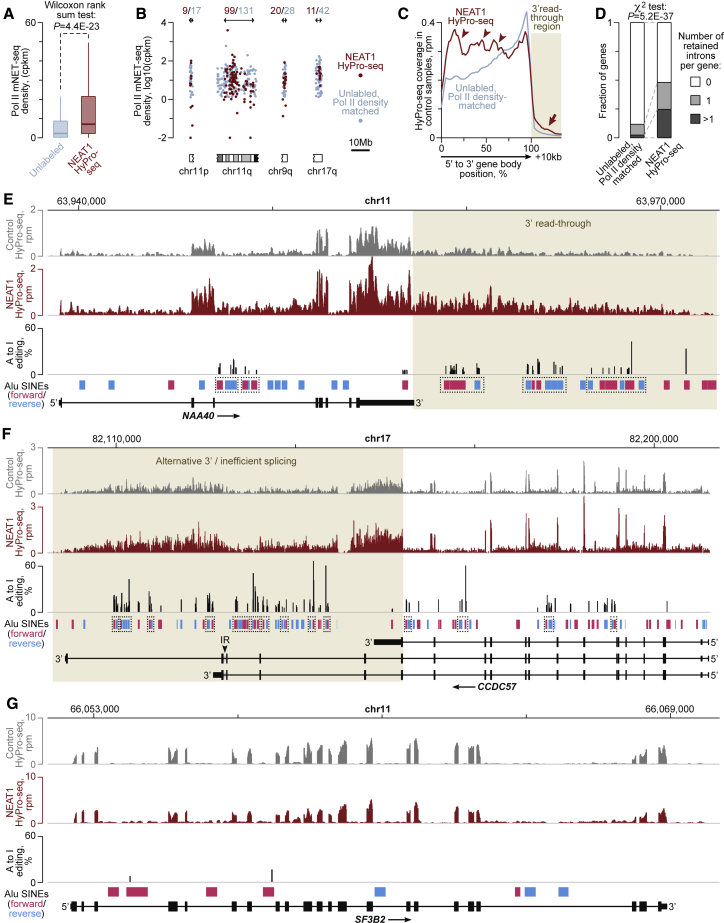
NEAT1 HyPro-labeled transcripts are often incompletely and A-to-I edited (A) Two-tailed Wilcoxon test analysis of mNET-seq coverage data (Nojima et al., 2015) showing that NEAT1-labeled genes tend to have higher density of elongating Pol II complexes compared with unlabeled but detectably expressed controls. (B) Red, genomic clusters of NEAT1 HyPro-seq targets; blue, a subset of their unlabeled neighbors with median Pol II density matched to that of the HyPro-seq genes. Note that many well-transcribed genes are not HyPro labeled. (C) Metagene plots showing that NEAT1 proximity-labeled transcripts contain 3′-read-through sequences (arrow) and gene-body peaks (arrowheads) possibly corresponding to retained introns more often than their unlabeled, Pol II density-matched counterparts. (D) NEAT1 HyPro targets are characterized by widespread retention of introns compared with unlabeled, Pol II density-matched controls. The incidence of introns retained in HyPro-labeled transcripts compared with the HyPro infusion control with > 10% ΔPSI and < 0.05 FDR IRFinder (Middleton et al., 2017) cutoffs was compared using the χ^2^ test. (E–G) Control and NEAT1 HyPro-seq coverage plots for NEAT-labeled targets (E) *NAA40* (chr11q) and (F) *CCDC57* (chr17) and (G) the unlabeled control *SF3B2* (chr11q). Note accumulation of NEAT1 HyPro-seq reads in the Pol II read-through region downstream of the 3′-terminal cleavage/polyadenylation site in *NAA40* and the poorly processed 3′-proximal region of *CCDC57* that may be also subject to alternative cleavage/polyadenylation. The two bottom tracks also show that the 3′-proximal regions of *NAA40* and *CCDC57* but not *SF3B2* harbor multiple inverted Alu SINEs undergoing extensive A-to-I editing. Dotted rectangles in (E) and (F) mark inverted Alu units edited at more than one position. The arrowhead in (F) shows the CCDC57 intron significantly retained in the HyPro-labeled sample compared with the HyPro infusion control (>10% ΔPSI and <0.05 FDR IRFinder cutoffs). See also Figures S5 and S6.

NEAT1 HyPro-seq genes also showed a higher density of Pol II-specific mNET-seq reads (Nojima et al., 2015) compared with their unlabeled counterparts (defined as genes with NEAT1 versus control fold enrichment < 1 and/or fold enrichment < 1.5 and FDR > 0.1; Figure 6A). However, even after selecting unlabeled genes with Pol II density matching that of the NEAT1-labeled distribution, the significant chr11q, chr11p, chr9q, and chr17q clusters contained more unlabeled than labeled targets (Figure 6B). This suggested that active transcription is necessary but not sufficient for RNA accumulation near paraspeckles.

In the search for the additional requirements, we compared control-labeled metagene plots for the NEAT1 targets and the Pol II density-matched unlabeled controls (Figure 6C). This revealed relatively high coverage of the NEAT1 targets in the first approximately two-thirds of the gene body and the ∼10 kb region downstream of the expected gene end. To test if the gene body peaks may correspond to introns retained in NEAT1-proximal transcripts, we analyzed differences in intron excision efficiency between NEAT1 and control HyPro-seq experiments using IRFinder (Middleton et al., 2017). Increased intron retention in the NEAT1 HyPro-seq samples (change in splicing efficiency ΔΨ > 10%, FDR < 0.05) was clearly over-represented among the NEAT1-labeled genes compared with the Pol II density-matched unlabeled controls (Figure 6D). This effect was even more apparent when we increased the stringency of the ΔΨ cutoff (Figure S5J).

Primary transcripts produced by Pol II often contain superfluous 3′ read-through sequences, which are subsequently removed by cleavage and polyadenylation (Nojima et al., 2015; Proudfoot, 2016). To find out if the increased metaplot coverage of the NEAT1 targets in the +10 kb downstream region (Figure 6C) could be due to inefficient 3′ end processing, we compared RPM-normalized HyPro-seq coverage plots for the NAA40 and CCDC57 targets and the unlabeled control SF3B2 (Figures 6E–6G). Although these three genes are transcribed with comparable efficiencies (average mNET-seq Pol II densities 9.3, 5.2, and 10.4 counts per million per kb, respectively), only NAA40 and CCDC57 but not SF3B2 showed dramatic accumulation of HyPro-seq reads in the 3′-terminal regions. The read-through coverage of NAA40 increased relative to the gene body in the NEAT1 HyPro-seq data compared with the HyPro infusion control (Figure 6E). CCDC57 showed generally poor 3′-terminal processing and significantly stronger retention of its penultimate intron in NEAT1 HyPro-seq versus HyPro infusion control (Figure 6F). Incomplete processing of NEAT1-labeled transcripts was also evident when we compared NEAT1 HyPro-seq with total RNA sequencing data (Figures S6A–S6C)

Transcribed 3′-proximal sequences of NAA40 and CCDC57 but not SF3B2 also contained numerous inverted short interspersed nuclear elements (SINEs) undergoing extensive A-to-I editing (detectable as A-to-G transitions; see STAR Methods for more detail) in NEAT1 HyPro-seq samples (Figures 6E–6G). As A-to-I-edited inverted SINEs have been reported to interact with paraspeckles (Anantharaman et al., 2016; Chen and Carmichael, 2009; Prasanth et al., 2005; Wang et al., 2018), we wondered if this was a common feature for the NEAT1 HyPro-seq targets. Notably, the incidence of inverted SINEs in the 3′ read-through region of this group of genes was significantly higher compared with their unlabeled counterparts (Figure S5K). At least some of these downstream elements were included into NEAT1-proximal transcripts and edited (Figures S6D–S6F).

Thus, NEAT1 HyPro-labeled transcripts tend to be incompletely processed and A-to-I edited.

### Relationship between paraspeckle proximity and gene expression

**Figure 7:**
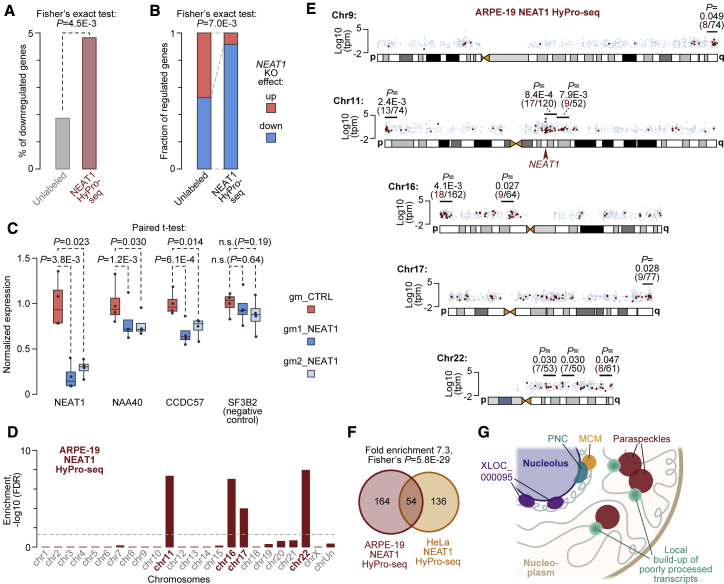
Relationship between paraspeckle proximity and gene expression (A) Fisher’s exact test analysis showing that NEAT1 HyPro-seq targets are significantly enriched for genes predicted to be downregulated (>1.5-fold, p < 0.05) in NEAT1-KO HeLa cells (Wang et al., 2018). (B) Downregulation is also a significantly over-represented type of gene response to NEAT1 KO among NEAT1 proximity-labeled targets. (C) qRT-PCR data showing that acute knockdown of NEAT1 by gapmers (gm1_NEAT1 or gm2_NEAT1) dampens the expression of NEAT1 proximity-labeled transcripts NAA40 and CCDC57 but not the SF3B2 control compared with the non-targeting gm_CTRL. Data from four independent transfection experiments carried out on different days were normalized to the expression of ACTB mRNA and corresponding control-gapmer mean values and compared using paired t test. (D) Chromosome enrichment pattern of NEAT1 HyPro-seq genes in ARPE-19 cells plotted as described in Figure 4A. (E) ARPE-19 NEAT1 targets also show statistically significant clustering on the four chromosomes enriched in (D) as well chr9q. The data are analyzed and plotted as in Figures 4B–4D. (F) HyPro-seq identifies a significantly overlapping but distinct set of NEAT-proximal targets identified in ARPE-19 compared with HeLa. (G) Spatial interactions identified in this study. See also Figure S7 and Table S5.

To address possible role of paraspeckles in regulation of its neighbors, we compared NEAT1 HyPro-seq data with transcriptome-wide effects of NEAT1 knockout (KO) in HeLa cells by CRISPR-Cas9 (Wang et al., 2018). Although the overlap between the two datasets was relatively small, we detected statistically significant enrichment of genes downregulated by NEAT1-KO with >1.5-fold change and p < 0.05 cutoffs among the NEAT1-HyPro-seq hits (Figures 7A and 7B).

Inactivation of NEAT1 was predicted to reduce the expression of *NAA40* (1.64-fold down, p = 0.037) and *CCDC57* (1.52-fold down, p = 0.0495) but not *SF3B2* (1.14-fold down, p = 0.48). To test this prediction, we treated HeLa cells with NEAT1-specific (gm1_NEAT1 or gm2_NEAT1) or non-targeting control (gm_CTRL) gapmer oligonucleotides and analyzed the samples using quantitative reverse transcriptase PCR (qRT-PCR). Both NEAT1-specific gapmers dampened the expression of NEAT1 compared with the non-targeting control and significantly downregulated *NAA40* and *CCDC57* but not *SF3B2* (Figure 7C). Notably, when we repeated the experiment using the more efficient gm1_NEAT1 and stained the samples using RNA-FISH with NEAT1- and target-specific probes, cells showing the strongest knockdown of NEAT1/paraspeckles were also characterized by the lowest expression of *NAA40* and *CCDC57* (Figure S7).

We finally wondered if paraspeckles might form spatially ordered contacts with transcripts originating from specific chromosomal regions in other cell types. To this end, we repeated NEAT1 HyPro-seq for the diploid retinal epithelial cell line ARPE-19 expressing paraspeckles at a readily detectable level (Figures S1G and S1L). Similar to HeLa, significantly labeled NEAT1 targets (>1.5-fold enrichment; FDR < 1E-10) were over-represented on chr11 and chr17 (Figure 7D) and clustered in a wide *NEAT1*-containing region of chr11q and telomere-proximal parts of chr9q chr11p, and chr17q (Figure 7E). Specific targets encoded on chromosomes enriched in the ARPE-19 or HeLa NEAT1 HyPro-seq experiments showed a significant overlap (Figure 7F; Table S5). However, there were also important differences likely reflecting distinct gene activity patterns of the two cell lines. For example, one of the two HeLa chr9q clusters was absent in ARPE-19, with several new clusters appearing on chr16 and chr22 (Figures 7D and 7E; Table S5).

Taken together, these data suggest that at least a subset of NEAT1 HyPro-seq genes require NEAT1/paraspeckles for optimal expression and the paraspeckle-proximal targets are overlapping but distinct between different cell types.

## Discussion

The HyPro technologies described in this work provide a valuable resource for understanding structure and functions of cellular RNA assemblies. Our labeling approach does not require genetic perturbations, extending its utility to a substantially wider range of cell types and organisms compared with live proximity-labeling methods. The use of fixed and permeabilized cells and a compact recombinant enzyme interacting with digoxigenin with a subnanomolar affinity eliminates possible artifacts caused by mislocalization and/or cytotoxicity of recombinant biotinylation enzymes in living cells. This may also reduce the time and efforts required to optimize labeling specificity and selectivity..

We show that HyPro-MS and HyPro-seq can identify molecular components both directly associated with and spatially proximal to RNA compartments of interest (e.g., Figures 2 and 3). A side-by-side comparison with genes short-listed by RIC-seq with the same stringency argues that HyPro-seq captures proximity patterns not necessarily depending on direct RNA-RNA interactions (Figures 4D and 4E; Figure S5G). This in turn appears to sensitize detection of long-range intrachromosomal and interchromosomal contacts between localized ribonucleoprotein complexes.

Data obtained for PNCTR, a lncRNA expressed at < 50 copies per cell (Yap et al., 2018), indicate that HyPro-MS and HyPro-seq are sufficiently sensitive to interrogate proximity networks of a wide range of RNA molecules of interest. We used 5 million to 10 million cells per sample throughout this study. Given the exceptionally high affinity of the biotin-streptavidin interaction and efficient solubilization of crosslinked material in our protocol, it may be possible to analyze RNA baits expressed at a lower level than PNCTR by simply scaling up the number of cells. Further gain in HyPro-seq sensitivity may be achieved by increasing the number of oligonucleotides per probe set and/or substituting biotin-phenol with an alternative labeling reagent, biotin-aniline (Zhou et al., 2019a).

Importantly, our work uncovers recurrent contacts between nuclear RNA compartments and other molecular structures (Figure 7G). Pointing at possible biological importance of such contacts, a top-scoring 45S HyPro-seq target, XLOC_000095, localizing to perinucleolar foci contains several regions of interspecies homology (Figure 3C). Of the nuclear (pre-)mRNA compartments uncovered by NEAT1 HyPro-seq, we show that at least the *NAA40* and *CCDC57* genes require NEAT1/paraspeckles for their optimal expression (Figure 7C; Figure S7). It will be also interesting to understand the mechanisms and possible significance of the proximity between PNCTR and the MCM complex involved in DNA replication (Figure 2I; Figure S3).

Combined analyses of HyPro-seq, CHART-seq, Hi-C, FISH, and mNET-seq data argue that (pre-)mRNAs accumulating near paraspeckles are likely newly synthesized and retained in the vicinity of their transcription sites (Figures 4, 5, and 6). We show that these transcripts differ from actively transcribed but non-compartmentalized controls by relatively inefficient splicing and 3′-terminal processing (Figures 6C–6G; Figures S5J and S6). We also detect a high incidence of transcribed and A-to-I-edited inverted SINE elements in the 3′-proximal regions of compartmentalized transcripts (Figures 6E–6G; Figures S5K and S6D–S6F). Although understanding functional significance of these findings will require further studies, it is clear that the ability of HyPro-seq to resolve RNA processing and editing events should expand the range of possible applications of this technology.

A significant fraction of paraspeckle-proximal targets identified in HeLa cells are also detected in the non-transformed epithelial cell line ARPE-19 (Figures 7D–7F). This includes transcripts from the interchromosomal gene clusters (e.g., CCDC57 and SNAPC4; Table S5) indicating that some paraspeckle-proximal targets are invariant across different cell types. Yet the numbers of non-overlapping targets are sufficiently large to propose that the structure of this interaction network is subject to regulation.

The paraspeckle compartment is known to undergo remodeling in response to different types of stress (Adriaens et al., 2016; Imamura et al., 2014; Mello et al., 2017; Wang et al., 2018). How such changes affect the repertoire of paraspeckle-proximal transcripts, their processing status and expression are important questions for future studies. At least in one published example, 3′-extended mouse CAT2/CTN transcripts localized to paraspeckles under normal conditions were cleaved in response to stress releasing translation-competent 5′-proximal fragments (Prasanth et al., 2005). Whether similar regulation mechanisms might operate in the case of human NEAT1 HyPro-seq targets is an exciting question for the future.

### Limitations

Our study introduces a versatile technology for unbiased proteomic and transcriptomic analyses of RNAs of interest in genetically unperturbed samples and sheds new light on the emerging relationship between RNA compartments, nuclear organization, and regulation of gene expression in eukaryotic cells. Yet, similar to other proximity biotinylation techniques, a key limitation of HyPro-MS and HyPro-seq is the relatively large labeling radius, expected to increase the incidence of false positives for smaller RNA compartments. This can be tackled, at least in part, by comparing proteomes and transcriptomes associated with the compartment of interest and control RNAs with similar intracellular localization (Figures S2C and S2D). Future improvements of HyPro-MS may involve designing digoxigenin-binding versions of other proximity-labeling enzymes that require direct protein contact for efficient biotinylation (Qin et al., 2021). It will be also interesting to see if our technology can be adapted for analysis of RNA-DNA proximity patterns (Chen et al., 2018).

Another possible limitation is the need to control for probe hybridization specificity. Although biotinylation in the no-probe and scrambled controls was negligible compared with probe sets against abundant transcripts (Figures S1J–S1L, S1P, and S1Q), unspecific labeling might become more problematic for rarer RNA targets. We therefore recommend validating specificity of newly designed probe sets by HyPro-FISH with appropriate negative and positive controls before attempting HyPro-MS and/or HyPro-seq analyses.

Finally, it is possible that digoxigenin-labeled oligonucleotide probes used in our method may compete with RNA-binding proteins interacting with overlapping target sequences. This in turn may interfere with probe binding and/or cause a partial loss of some target-proximal molecules. It is reassuring in this regard that the (UC)n-specific PNCTR probe successfully identified the PTBP1 protein known to interact with the (UC)n sequences (Table S2; Yap et al., 2018). However, repeating HyPro-labeling with more than one probe set against the same RNA target transcript (Figure 2C) should be considered for improved detection sensitivity and specificity.

## STAR★Methods

Detailed methods are provided in the online version of this paper and include the following:

### Key resources table

**Table:** 

REAGENT or RESOURCE	SOURCE	IDENTIFIER
**Antibodies**		

SEEBRIGHT® Red 650 dUTP	Enzo Life Sciences	Cat#: ENZ-42522
SEEBRIGHT® Green 496 dUTP	Enzo Life Sciences	Cat#: ENZ-42831
Streptavidin-HRP	Thermo Fisher Scientific	Cat#: SA10001
Rabbit anti-Fibrillarin	Abcam	Cat#: ab5821; RRID:AB_2105785
Rabbit anti-hnRNPL	Abcam	Cat#: ab32680; RRID:AB_941986
Rabbit anti-MCM5	Abcam	Cat#: ab75975; RRID:AB_1310439
Rabbit anti-MCM2	Abcam	Cat#: ab95361; RRID:AB_10679573
Rabbit anti-SFPQ	Abcam	Cat#: 38148; RRID:AB_945424
Rabbit anti-PTBP1	Abcam	Cat#: ab133734; RRID:AB_2814646
Mouse anti-Digoxigenin	Jackson Laboratories	Cat#: 200-002-156; RRID:AB_2339005
Alexa Fluor647-conjugated anti-mouse IgG (H+L)	ThermoFisher Scientific	Cat#: A31571; RRID:AB_162542
Alexa Fluor647-conjugated streptavidin	Biolegend	Cat#: 405237

**Bacterial and virus strains**		

TOP10 *E. coli*	Thermo Fisher Scientific	Cat#: C404010
SoluBL21 *E. coli*	Amsbio	Cat#: C700200

**Chemicals, peptides, and recombinant proteins**		

IPTG	Promega	Cat#: V3951
BugBuster protein extraction reagent	Millipore	Cat#: 70584
rLysozyme	Millipore	Cat#: 71110
Benzonase	Millipore	Cat#: 70664
Vitronectin, truncated recombinant human (VTN-N)	Thermo Fisher Scientific	Cat#: A14700
Dimethyl sulfoxide (DMSO)	Sigma Aldrich	Cat#: D2650
TRIzol LS reagent	Thermo Fisher Scientific	Cat#: 10296010
Purelink DNase set	Thermo Fisher Scientific	Cat#: 12185010
TURBO DNase	Thermo Fisher Scientific	Cat#: AM2238
RNase inhibitor, murine	New England Biolabs	Cat#: M0314
SuperScript IV reverse transcriptase	Thermo Fisher Scientific	Cat#: 18090200
Formaldehyde	Thermo Fisher Scientific	Cat#: 28908
DSP (dithiobis(succinimidyl propionate))	Thermo Fisher Scientific	Cat#: 22585
Salmon sperm DNA	Thermo Fisher Scientific	Cat#: 15632011
Human Cot-1 DNA	Thermo Fisher Scientific	Cat#: 15279011
Yeast tRNA	Thermo Fisher Scientific	Cat#: AM7119
20x SSC	Thermo Fisher Scientific	Cat#: AM9763
DAPI	Thermo Fisher Scientific	Cat#: D1306
Biotin-phenol	Caltag Medsystems	Cat#: CDX-B0270
Hydrogen peroxide	Sigma Aldrich	Cat#: H1009
Trolox	Sigma Aldrich	Cat#: 238813
Sodium ascorbate	Sigma Aldrich	Cat#: A4034
cOmplete, EDTA-free protease inhibitor cocktail	Sigma Aldrich	Cat#: 4693132001
PMSF	Cell Signaling Technology	Cat#: 8553
MyOne streptavidin C1 magnetic beads	Thermo Fisher Scientific	Cat#: 11205D
Ammonium bicarbonate	Sigma Aldrich	Cat#: 09830
Water for chromatography (LC-MS Grade)	Merck	Cat#: 1153331000
Trypsin / Lys-C Mix	Promega	Cat#: V5073

**Critical commercial assays**		

Pierce BCA Protein Assay kit	Thermo Fisher Scientific	Cat#: 23225
Enhanced Chemiluminescence (ECL) substrate	Thermo Fisher Scientific	Cat#: 32109
Immobilon Chemiluminescent HRP (ECL) substrate	Millipore	Cat#: WBKLS0500
Peroxidase Activity Assay kit	Sigma Aldrich	Cat#: MAK092
DIG Oligonucleotide 3′ End Labeling kit	Sigma Aldrich	Cat#: 03353575910
Purelink RNA mini kit	Thermo Fisher Scientific	Cat#: 12183018A
RNA Clean & Concentrator kit	Zymo Research	Cat#: R1015
NEBNext® rRNA Depletion kit	New England Biolabs	Cat#: E6350
NEBNext Ultra II Directional RNA library Prep kit for Illumina and barcoded primers	New England Biolabs	Cat#: E7765
NGSBIO Library Quant Kit Blue	PCR Biosystems	Cat# PB71.15-01
Macherey-Nagel NucleoBond BAC 100 kit	Thermo Fisher Scientific	Cat#: 12768482
Nick translation DNA labeling system 2.0	Enzo Life Science	Cat#: ENZ-GEN111-0050

**Deposited data**		

APEX-seq analysis of HEK293T cells, sample GSM3206948	Fazal et al., 2019	https://www.ncbi.nlm.nih.gov/geo/query/acc.cgi?acc=GSM3206948
APEX-seq analysis of HEK293T cells, sample GSM3206949	Fazal et al., 2019	https://www.ncbi.nlm.nih.gov/geo/query/acc.cgi?acc=GSM3206949
APEX-seq analysis of HEK293T cells, sample GSM3206950	Fazal et al., 2019	https://www.ncbi.nlm.nih.gov/geo/query/acc.cgi?acc=GSM3206950
APEX-seq analysis of HEK293T cells, sample GSM3206951	Fazal et al., 2019	https://www.ncbi.nlm.nih.gov/geo/query/acc.cgi?acc=GSM3206951
RNA-seq analysis of HeLa cells with genetically inactivated NEAT1, sample GSM3016459	Wang et al., 2018	https://www.ncbi.nlm.nih.gov/geo/query/acc.cgi?acc=GSM3016459
RNA-seq analysis of HeLa cells with genetically inactivated NEAT1, sample GSM3016460	Wang et al., 2018	https://www.ncbi.nlm.nih.gov/geo/query/acc.cgi?acc=GSM3016460
RNA-seq analysis of HeLa cells with genetically inactivated NEAT1, sample GSM3016461	Wang et al., 2018	https://www.ncbi.nlm.nih.gov/geo/query/acc.cgi?acc=GSM3016461
RIC-seq analysis of HeLa cells, sample GSM3629915	Cai et al., 2020	https://www.ncbi.nlm.nih.gov/geo/query/acc.cgi?acc=GSM3629915
RIC-seq analysis of HeLa cells, sample GSM3629916	Cai et al., 2020	https://www.ncbi.nlm.nih.gov/geo/query/acc.cgi?acc=GSM3629916
mNET-seq analysis of HeLa cells, sample GSM2357382	Nojima et al., 2015	https://www.ncbi.nlm.nih.gov/geo/query/acc.cgi?acc=GSM2357382
HyPro-seq analysis of HeLa and ARPE-19 cells	This study	ArrayExpress: E-MTAB-10365
HyPro-MS analysis of HeLa cells	This study	ProteomeXchange Consortium: PXD025264; 10.6019/PXD025264

**Experimental models: Cell lines**		

HeLa	ATCC	Cat#: CCL-2
APRE-19	ATCC	Cat#: CRL-2302
iPSC	HipSci	HPSI0314i-cuhk_1

**Oligonucleotides**		

Antisense LNA Gapmer, negative control A (gmControl) (5′A^∗^A^∗^C^∗^A^∗^C^∗^G^∗^T^∗^C^∗^T^∗^A^∗^T^∗^A^∗^C^∗^G^∗^C)	Exiqon	Cat#: 339516/LG00000002
gm1_NEAT1(5′-T^∗^A^∗^A^∗^G^∗^C^∗^A^∗^C^∗^T^∗^T^∗^T^∗^G^∗^G^∗^A^∗^A^∗^A^∗^G-3′)	Exiqon	Cat#: 339511/LG00196607-DDA
gm2_NEAT1 (5′- C^∗^T^∗^C^∗^A^∗^C^∗^A^∗^C^∗^G^∗^T^∗^C^∗^C^∗^A^∗^T^∗^C^∗^T-3′)	Exiqon	Cat#: 339511/LG00169935-DDA
NEAT1-specific Quasar 570-labeled Stellaris® probe set	Biosearch Technologies	Cat#: SMF-2036-1
PNCTR-specific Quasar 670-labeled Stellaris® probe set	Biosearch Technologies	Same sequences as in Table S1, PNCTR(NR)
DNA oligonucleotide probes used for HyPro-FISH or RNA-FISH	IDT	See Table S1 for more detail

**Recombinant DNA**		

pET28a (cloning vector)	Clontech	Cat#: 69864-3
pML433 (plasmid for expressing HyPro enzyme in *E. coli*)	This study	N/A
BAC RP11-456L5 (NEAT1)	BACPAC Genomics	https://bacpacresources.org/
BAC RP11-326I13 (INPPL1)	BACPAC Genomics	https://bacpacresources.org/
BAC RP11-475F12 (CCDC57)	BACPAC Genomics	https://bacpacresources.org/
BAC RP11-707O3 (SNAPC4)	BACPAC Genomics	https://bacpacresources.org/
BAC RP11-956G20 (IFNA gene cluster)	BACPAC Genomics	https://bacpacresources.org/

**Software and algorithms**		

HISAT2, version 2.1.0	Pertea et al., 2016	http://daehwankimlab.github.io/hisat2/manual/
StringTie, version 2.1.4	Pertea et al., 2016	https://ccb.jhu.edu/software/stringtie/
Trimmomatic, version 0.38	Bolger et al., 2014	www.usadellab.org/cms/?page=trimmomatic
BedTools, version 2.29.0	Quinlan and Hall, 2010	https://bedtools.readthedocs.io/en/latest/
SAMtools, version 1.7	Li et al., 2009	http://www.htslib.org/
Kallisto, version 0.46.0	Bray et al., 2016	https://pachterlab.github.io/kallisto/about
Proteome Discoverer, version 2.2	Thermo Fisher Scientific	Cat#: OPTON-30811
Scaffold, version 4.11.0	Proteome Software, Inc	http://www.proteomesoftware.com/products
R, version 4.1.0	R Core Team, 2019	https://www.r-project.org/
Bioconductor, version 3.13	https://bioconductor.org/	https://bioconductor.org/install/
LightCycler 96 software, version 1.1.0.1320	Roche	https://pim-eservices.roche.com/eLD/web/pi/en/documents/download/861207d6-aecd-ea11-fe90-005056a772fd
ZEN Blue, version 2.5	ZEISS	https://www.zeiss.com/microscopy/int/products/microscope-software.html
Fiji, version 1.53c	https://fiji.sc/	https://imagej.net/software/fiji/downloads
Image Studio Lite, version 5.2	LI-COR Biotechnology	https://www.licor.com/bio/image-studio-lite/

**Other**		

Detailed HyPro labeling protocol	This study	Methods S1

### Resource availability

#### Lead contact

Further information and request for resources and reagents should be directed to the lead contact, Eugene V. Makeyev (eugene.makeyev@kcl.ac.uk).

#### Materials availability

Commercially available reagents are listed in the key resources table. The pML433 plasmid for expression of soluble HyPro protein in bacteria will be made publicly available through Addgene.

### Experimental model and subject details

#### Cell culture

HeLa cells (ATCC®CCL-2) and ARPE-19 (ATCC®CRL-2302) were cultured in a humidified incubator at 37°C, 5% CO2, in DMEM containing 4.5 g/L glucose, GlutaMAX and 110 mg/L sodium pyruvate (Thermo Fisher Scientific, cat# 11360070) supplemented with 10% FBS (Hyclone, cat# SV30160.03) and 100 units/ml PenStrep (Thermo Fisher Scientific, cat# 15140122). For passaging, cells were washed with 1 × PBS and dissociated in 0.05% Trypsin-EDTA (Thermo Fisher Scientific, cat# 15400054) for 5 min at 37°C. In gapmer transfection experiments, HeLa cells were seeded at 0.6 × 10^5^ per well of a 12-well plate overnight. The next day, 25 nM gm_CTRL (QIAGEN, cat# 339516/LG00000002, 5′-A^∗^A^∗^C^∗^A^∗^C^∗^G^∗^T^∗^C^∗^T^∗^A^∗^T^∗^A^∗^C^∗^G^∗^C-3′), gm1_NEAT1(5′-T^∗^A^∗^A^∗^G^∗^C^∗^A^∗^C^∗^T^∗^T^∗^T^∗^G^∗^G^∗^A^∗^A^∗^A^∗^G-3′) or gm2_NEAT1 (5′- C^∗^T^∗^C^∗^A^∗^C^∗^A^∗^C^∗^G^∗^T^∗^C^∗^C^∗^A^∗^T^∗^C^∗^T-3′) in 50 μl of OPTI-MEM I (Thermo Fisher Scientific, cat# 31985047) were mixed with 2 μl Lipofectamine 3000 (Thermo Fisher Scientific, cat# L3000008) pre-diluted in 50 μl of OPTI-MEM I and incubated for 15 min at room temperature. The mixture was added drop-wise to the cells followed by a 48-hour incubation at 37°C, 5% CO2 and RT-qPCR or RNA-FISH analyses (see below).

Human induced pluripotent stem cells (HipSci, HPSI0314i-cuhk_1) were cultured in Essential 8 (Thermo Fisher Scientific, cat# A1517001) supplemented with 100 units/ml PenStrep (Thermo Fisher Scientific, cat# 15140122) on plates coated with 1 μg/cm^2^ VTN-N (Thermo Fisher Scientific, cat# A14700) in a humidified incubator at 37°C, 5% CO2. For passaging, cells were washed with DPBS (no calcium, no magnesium; Thermo Fisher Scientific, cat# 14190094), incubated with 0.5 mM EDTA (Thermo Fisher Scientific, cat# 15575020) for 7 min at room temperature, and gently triturated in Essential 8 media.

### Method details

#### RT-qPCR

Total cellular RNAs were isolated using TRIzol (Thermo Fisher Scientific), as recommended, with an additional 10-min incubation at 50°C prior to separating the phases with chloroform. RNA was precipitated from the aqueous phase with an equal volume of isopropanol, washed with 70% ethanol and rehydrated in 80 μL of nuclease-free water. RNA samples were then treated with 4 units of Turbo DNase (Thermo Fisher Scientific, cat# AM2238) at 37°C for 30 min to remove traces of genomic DNA, extracted once with an equal volume of acidic phenol-chloroform (1:1) and chloroform, and precipitated with 3 volumes of 100% ethanol and 0.1 volume of 3 M sodium acetate (pH 5.2), washed with 70% ethanol, and dissolved in nuclease-free water (Thermo Fisher Scientific, cat# AM9939). The RNAs were then reverse-transcribed (RT) using SuperScript IV reagents (Thermo Fisher Scientific, cat# 18090200) for 40 min at 50°C. The reactions were then analyzed by quantitative PCR using a Light Cycler®96 Real-Time PCR System (Roche), qPCR BIO SyGreen Master Mix (PCR Biosystems; cat# PB20.16), and the following primers:

Neat1_F (MLO2752) 5′-GTATGCAGCTTGGCACTGG-3′
Neat1_R (MLO2753) 5′-GGCTCACCCACGCACTAA-3′
CCDC57_F (MLO3343) 5′-AAGCTGGCCCAGTATCCTCT-3′
CCDC57_R (MLO3344) 5′-TGGGCTATCCTGAGATGCTT-3′
NAA40_F (MLO3272) 5′-CAATCATGGTGCCTACCAGTT-3′
NAA40_R (MLO3273) 5′-CTGAGAGTTCAGTGGCAGCA-3′
SF3B2_F (MLO3100) 5′-GCAGCTGATGTTGAGATTGAGT-3′
SF3B2_R (MLO3101) 5′-TGTGCTCCTCTTCAAATCCCT-3′
ACTB_F (MLO572) 5′-CATGTACGTTGCTATCCAGGC-3′
ACTB_R (MLO573) 5′-CTCCTTAATGTCACGCACGAT-3′

RT-qPCR signals were normalized to the expression level of the ACTB “housekeeping” mRNA.

#### Recombinant HyPro enzyme

To prepare the HyPro expression construct pEML433, a synthetic DNA fragment (gBlock, IDT) encoding APEX2 and DIG10.3 protein sequences fused by a flexible linker was cloned into pET28a (Clontech) at XhoI-XbaI (New England Biolabs). SoluBL21 *E. coli* cells (Amsbio) transformed with pEML433 were grown overnight in LB broth (VWR) with 25 μg/ml kanamycin at 37°C with shaking at 250 rpm. Four ml of the overnight culture was diluted with 600 mL of fresh LB broth with 25 μg/ml kanamycin and the shaking was continued in a 2-l flask at 37°C until OD600 = 0.6 (∼3 hours). The culture was then chilled on ice for 10 min, supplemented with 1 mM Isopropyl β-D-1-thiogalactopyranoside (IPTG, Promega), and shaken for another 24 hours at 25°C to express the HyPro protein. Cells were collected by centrifugation at 10,000 × g for 10 min at 4°C. The bacterial pellet was resuspended in 45 mL of BugBuster protein extraction reagent (Merck Millipore) supplemented with 1500 units/ml rLysozyme (Merck Millipore) and 25 units/ml benzonase (Merck Millipore) and incubated at room temperature for 30 min with constant rotation. The lysate was clarified by centrifugation at 16,000 × g for 20 min at 4°C. The supernatant was filtered through a 0.45-μm low-protein binding syringe filter and loaded onto two sequentially connected 1 mL HisTrap FF Crude Columns (GE Healthcare) equilibrated with buffer A (20 mM Tris pH 8.0, 100 mM NaCl, 25 mM imidazole, 14 mM beta-mercaptoethanol (β-ME)). The column was then washed with 20 mL of buffer A and the protein was step-eluted with 50% of buffer B (20 mM Tris pH 8.0, 100 mM NaCl, 500 mM imidazole, 14 mM β-ME). The eluted fraction was then loaded onto a HiLoad 26/60 Superdex 75 column (GE Healthcare) equilibrated with buffer C (20 mM Tris, pH 8.0, 100 mM NaCl, 1 mM DTT). Protein elution was monitored by UV absorbance at 280 nm and measuring protein concentration in fractions using a Pierce BCA Kit (Thermo Fisher Scientific, cat# 23225), as recommended. Fractions containing the highest concentration of the HyPro protein and lacking major contaminating protein bands according to SDS-PAGE/Coomassie R-250 were combined, aliquoted, snap-frozen in liquid nitrogen, and stored at −80°C for up to a year.

#### Peroxidase assays

For a rapid semiquantitative test of peroxidase activity, 1-μl protein samples were mixed with 20 μL of reconstituted Enhanced Chemiluminescence (ECL) substrate (Thermo Fisher Scientific, cat# 32109), incubated for 1 min, spotted onto a piece of filter paper, and immediately imaged using an Odyssey FC system (LI-COR). Bovine serum albumin (BSA) was used as a negative control. We also quantified specific activity of purified HyPro protein samples using a Peroxidase Activity Assay Kit from Merck/Sigma-Aldrich (cat# MAK092). H_2_O_2_ standard curve and serially diluted HyPro reactions were set up in 96-well plates (Starlab, cat# E2996-1600) in principle as recommended by Merck/Sigma-Aldrich. The standard curve reactions were incubated at room temperature (22°C), followed by measuring the absorbance in a plate reader (Thermo Fisher Scientific) at 560 nm. HyPro protein reactions were incubated at room temperature and the absorbance was measured at 1, 3, 6, 10, 20, and 30 min time points. The linear part of the time course curve was used to calculate specific peroxidase activity of purified HyPro protein preps.

#### Digoxigenin binding assays

Digoxigenin-labeled RNAs and DNAs prepared by *in vitro* transcription (Makeyev et al., 2007) or nick translation (Yap et al., 2018), respectively, were spotted onto 0.45-μm nitrocellulose membrane (Sigma Aldrich, cat# GE10600016) and UV crosslinked at 120 mJ/cm^2^ (Stratalinker). The membrane was rinsed with 1 × TBST (20 mM Tris-HCl, pH 7.6, 150 mM NaCl, 0.2% Tween 20) and blocked with 5% BSA in 1 × TBST at room temperature for 1 hour. It was subsequently incubated with HyPro protein diluted in 1 × TBST and 1% BSA at room temperature for 1 hour followed by three washes with 1 × TBST. The membrane was then soaked in reconstituted Immobilon ECL reagent (Millipore, cat# WBKLS0500) and imaged using an Odyssey FC system (LI-COR).

#### DNA probes for proximity labeling, RNA-FISH and DNA-FISH

DNA oligonucleotide probes (Table S1) complementary to 45S, NEAT1, PNCTR, and their HyPro-seq targets were designed using Stellaris® probe designer program (LGC Biosearch Technologies, v4.2), purchased from IDT and labeled using a 2nd generation DIG Oligonucleotide 3′ End Labeling Kit (Sigma Aldrich) to yield 5 μM digoxigenin-labeled mixtures. For intron-containing genes, the mixtures contained equal numbers of exonic and intronic probes. The (GA)_10_ DNA oligonucleotide probe complementary to PNCTR (UC)_n_ repeats was ordered from IDT with a 3′-terminal digoxigenin modification (/3Dig_N/). In some RNA-FISH experiments, we used a NEAT1-specific Quasar 570-labeled Stellaris® probe set (Biosearch Technologies, cat# SMF-2036-1) and PNCTR-specific Quasar 670-labeled Stellaris® probe set (Biosearch Technologies; designed as described in Table S1).

To prepare DNA-FISH probes, bacterial artificial chromosomes (BACs) were purified with Macherey-Nagel NucleoBond BAC 100 Kit (Thermo Fisher Scientific, cat# 12768482) and labeling using a Nick Translation mix (Enzo, cat# ENZ-GEN111) and either SEEBRIGHT® Red 650 dUTP (ENZO ENZ-42522) or SEEBRIGHT® Green 496 dUTP (ENZO ENZ-42831) for 4 hours at 16°C. For each labeling experiment, 50 ng of nick-translated probe was precipitated with 5 μg salmon sperm DNA (Thermo Fisher Scientific, cat# 15632011), 2.5 μg human Cot-1 DNA (Thermo Fisher Scientific, cat# 15279011), 5 μg yeast tRNA (Thermo Fisher Scientific, cat# AM7119), 1/10 volume of 3 M sodium acetate, pH 5.5 and 3 volumes of 100% ethanol. The pellets were washed with 70% ethanol, dissolved in 10 μl formamide, denatured at 75°C for 10 min, mixed with 10 μl of 2 × hybridization buffer (0.4% BSA, 4 × SSC, 20% dextran sulfate), and pre-incubated at 37°C for 20 minutes prior to hybridization.

#### Proximity labeling *in situ*

Cells grown in 10- or 15-cm dishes (∼5-10 × 10^6^) or 12-well plates (∼1 × 10^5^-2 × 10^5^ per well) were fixed with 0.5 mg/ml dithiobis(succinimidyl propionate) (DSP; Thermo Fisher Scientific, cat# 22585) in 1 × PBS for 30 min at room temperature. The samples were then washed three times with 1 × PBS and 20 mM Tris-HCl, pH 8.0, 5 min each wash, permeabilized with 70% ethanol at room temperature for 1 hour, equilibrated in 2 × SSC and 10% formamide (Thermo Fisher Scientific) for 5 min, and hybridized with digoxigenin-labeled oligo probes in 2 × SSC, 10% formamide and 10% dextran sulfate overnight at 37°C. We used standardized hybridization and washing conditions, with probe concentrations adjusted according to RNA “bait” abundance detected in preliminary HyPro-FISH experiments: 45S-specific probe set, 5 nM; NEAT1-specific probe set, 25 nM; probe set against non-repetitious PNCTR sequences, 25 nM; probe against PNCTR (UC)n repeats, 50 nM. Following the hybridization, samples were washed with 2 × SSC and 10% formamide at 37°C for 30 min and 1 × SSC at room temperature for 15 min and blocked with 0.8% BSA in 4 × SSC (HyPro blocking buffer) and 100 units/ml murine RNase inhibitor (New England Biolabs, cat# M0314) at room temperature for 30 min.

We then incubated the samples with 2.7 μg/ml HyPro enzyme in HyPro blocking buffer at room temperature for 1 hour. Unbound HyPro was washed off with 4 × SSC, 4 × SSC and 0.1% Triton X-100, 4 × SSC for 10 min each, and left in 1 × PBS for 5 min. In HyPro infusion controls, cells were bathed in 1 × PBS containing 5.4 μg/ml of HyPro enzyme for 5 min. Proximity biotinylation was then carried out by the addition of an equal volume of 1 × PBS containing 1 mM biotin-phenol (Caltag Medsystems, cat# CDX-B0270) and 0.2 mM hydrogen peroxide (Sigma Aldrich, cat# H1009) and gently agitating the samples for 1 min. The reaction was quenched by quickly rinsing the samples three times with 5 mM Trolox (Sigma Aldrich, cat# 238813) and 10 mM sodium ascorbate (Sigma Aldrich, cat# A4034) in 1 × PBS. Samples labeled in dishes were then analyzed by immunoblotting, mass-spectrometry or RNA sequencing. The coverslips were used for HyPro-FISH.

#### Purification of biotinylated RNAs

To extract RNA from HyPro-labeled samples, cells were lysed in high-SDS RIPA buffer (150 mM NaCl, 1 mM EDTA, 50 mM Tris-HCl, pH 8.0, 1% NP-40, 0.5% sodium deoxycholate and 0.5% SDS) supplemented with 10 mM sodium ascorbate, 5 mM Trolox, 50 mM DTT and 100 units/ml of murine RNase inhibitor. Cells were incubated in 1 ml/10-cm or 2 ml/15-cm dish of this buffer for 10 min on ice, scraped off the plates and further incubated for 10 min on ice. The samples were then sonicated using a Bioruptor system (Diagenode) equipped with a refrigerated ice bath, for 5-10 cycles of 30 s ON / 30 s OFF at the HIGH power setting. The lysates were then incubated for 30 min at 37°C to reverse crosslinks, supplemented with 400 μg of proteinase K (Thermo Fisher) and incubated for 1 hour at 50°C. Three volumes of TRIzol LS (Thermo Fisher Scientific) was then added to the lysate, the phases were separated by chloroform, and total RNA was purified from the aqueous phase using Purelink RNA miniprep kit (Thermo Fisher), as recommended.

To capture biotinylated RNA, we used 10 μL of MyOne streptavidin C1 magnetic beads (Thermo Fisher Scientific, cat# 11205D) per 20 μg of total RNA eluted in in nuclease free-water. The beads were pre-washed three times in the B&W buffer (5 mM Tris-HCl, pH 7.5, 5 mM EDTA, pH 8.0, 1 M NaCl, 0.1% Tween 20), once with solution A (100 mM NaOH and 50 mM NaCl), once with solution A plus 0.1% Tween 20, once with 100 mM NaCl, and once with 100 mM NaCl, 10 mM Tris-HCl, pH 7.5, 1 mM EDTA, pH 8.0, 0.2% Tween 20. The beads were then resuspended in 150 μl of 100 mM NaCl, 10 mM Tris-HCl, pH 7.5, 1 mM EDTA, pH 8.0, 0.2% Tween 20 and incubated with an equal volume of total RNA for 2 hours at 4°C with continuous rotation. The beads were collected using a DynaMag-2 Magnet (Thermo Fisher), washed three times with B&W buffer and resuspended in 100 μl of 100 mM NaCl, 50 mM Tris-HCl, pH 7.5, 1 mM EDTA, 1% SDS and 0.80 μg/μl proteinase K. The beads were then incubated in a thermomixer at 50°C for 45 min and mixed with 300 μl of TRIzol LS and 80 μl of chloroform. Biotinylated RNAs were then purified from the aqueous phase using an RNA Clean & Concentrator kit (Zymo Research, cat# R1015).

#### Purification of biotinylated proteins

HeLa cells HyPro-labeled in 10-cm dishes were lysed with 600 μL of the high-SDS RIPA buffer supplemented with 10 mM sodium ascorbate, 5 mM Trolox, 50 mM DTT, cOmplete, EDTA-free protease inhibitor cocktail (Sigma Aldrich, cat# 4693132001), and 1 mM phenylmethanesulfonyl fluoride (PMSF, Cell Signaling Technology, cat# 8553), incubated on ice for 10 min and scraped off from the plates, and incubated for another 10 min on ice. The samples were then sonicated as described above and de-crosslinked by incubating the mixtures at 37°C for 1 hour. The lysates were clarified by centrifugation at 15,000 × g for 10 min at 4°C, transferred to fresh tubes and stored at −80°C until needed.

Sixty μl of MyOne streptavidin C1 magnetic beads were pre-washed twice with the RIPA buffer (150 mM NaCl, 1 mM EDTA, pH 8.0, 50 mM Tris-HCl, pH 8.0, 1% NP-40, 0.5% sodium deoxycholate, and 0.1% SDS), resuspended in 3 mL of RIPA buffer, combined with de-crosslinked lysates and incubated for 2 hours at room temperature as 3 separate aliquots. The beads were collected using a DynaMag-2 Magnet and washed twice with RIPA buffer, once with 1 M KCl, once with 0.1 M Na_2_CO_3_, once with 2 M urea in 10 mM Tris-HCl, pH 8.0 and twice with the RIPA buffer to remove unspecifically bound proteins. The beads were collected using DynaMag-2 Magnet and analyzed by immunoblotting or mass-spectrometry as described below.

#### RNA sequencing

Purified biotinylated transcripts were depleted of mature ribosomal RNAs using a NEBNext® rRNA Depletion Kit (New England Biolabs, cat# E6350), and used to prepare stranded sequencing libraries with a NEBNext Ultra II Directional RNA library Prep kit for Illumina and barcoded primers (New England Biolabs, cat# E7765) according to the manufacturer’s instruction. The barcoded libraries were quantified using NGSBIO Library Quant Kit Blue for Illumina® (PCR Biosystems, cat# PB71.15-01) and pooled prior to sequencing. Single-read sequencing was performed at the Oxford Genomics Centre, UK using a NextSeq 500 platform (Illumina, NextSeq 500/550 v2.5 Kits, 75 cycles) at ∼20 million reads per demultiplexed sample per single lane of the NextSeq 500 flow cell.

#### Immunoblotting

Protein-loaded beads were incubated with RIPA buffer supplemented with cOmplete EDTA free protease inhibitor cocktail, 1 mM PMSF and 50 mM DTT for 20 min at 37°C with gentle agitation. Proteins were eluted from the beads by adding an equal volume of 4 × LDS sample buffer (Life Technologies), 50 mM DTT and 5 mM biotin. This was then incubated at 70°C for 10 min. The eluates were analyzed by SDS-PAGE in 4%–12% NuPAGE Bis-Tris gels (Thermo Fisher Scientific, cat# NP0321) and electrotransferred to nitrocellulose membranes using Trans-Blot Turbo Transfer System (Bio-Rad) as recommended. The membranes were blocked in 1 × TBST and 5% BSA for 1 hour at room temperature and incubated for another hour with streptavidin-HRP (Thermo Fisher Scientific, cat# SA10001; diluted 1:20,000 in 1 × TBST and 5% BSA). Following three 5-munute washes with 1 × TBST, biotinylated proteins were detected using an ECL kit (Thermo Fisher Scientific, cat# 32109) and an Odyssey imaging system (Li-COR Biosciences).

#### Label-free mass-spectrometry

Protein-loaded beads were washed three times with 50 mM ammonium bicarbonate, pH 8.0 and resuspended in 45 μL 50 mM ammonium bicarbonate, pH 8.0 containing 1.5 μg of Trypsin/Lys-C protease mix (Promega). On-bead proteolysis was performed by incubating the suspension at 37°C overnight, with agitation. The next day, an additional 0.75 μg of Trypsin/Lys-C in 15 μL of 50 mM ammonium bicarbonate, pH 8.0 was added to the suspension and the incubation was continued for another 4 hours at 37°C. The beads were collected using a DynaMag-2 Magnet and the hydrolyzates were transferred to fresh microfuge tubes. Beads were washed twice with 45 μL aliquots of mass-spectrometry grade water and the two washes were combined with the original supernatants bringing the final volume to ∼150 μL and the concentration of ammonium bicarbonate to ∼20 mM. The samples were cleared by centrifugation at 16,000 × g for 10 min at 4°C and transferred to fresh tubes. Three replicated samples were analyzed for all conditions.

The subsequent sample preparation and label-free mass spectrometry steps were performed by the CEMS Proteomics Core Facility at King’s College London, UK. Peptides were purified using Pierce C18 spin columns (Thermo Fisher Scientific, UK) as recommended, eluted in 70% acetonitrile and dried in a SpeedVac (Thermo Fisher Scientific, UK). The samples were then resuspended in 2% acetonitrile in 0.05% formic acid (both Fisher Scientific, UK) and analyzed by LC-MS/MS. Chromatographic separation was performed using a U3000 UHPLC NanoLC system (Thermo Fisher Scientific, UK). Peptides were resolved by reversed-phase chromatography on a 50 cm-long 75 μm I.D. C18 Pepmap column using a linear gradient formed by buffers A (0.1% formic acid) and B (80% acetonitrile in 0.1% formic acid). The gradient was delivered at a flow rate of 250 nl/min, starting at 5% B (0-5 minutes), gradually increasing the percent of B to 40% (5-40 minutes), 99% B wash (40-45 minutes), and re-equilibrating the column at 5% B (45-60 minutes).

The eluates were ionised by electrospray ionisation using an Orbitrap Fusion Lumos Tribrid mass spectrometer (Thermo Fisher Scientific, UK) controlled by Xcalibur v4.1.5 software. The instrument was first programmed to acquire in data-dependent mode using a ‘universal’ Orbitrap-Ion Trap method by defining a 3 s cycle time between a full MS scan and MS/MS fragmentation. Orbitrap spectra (FTMS1) were collected at a resolution of 120,000 over a scan range of m/z 375-1500 with an automatic gain control (AGC) setting of 4 × 10^5^ and maximum injection time of 35 ms. Monoisotopic precursor ions were filtered using charge state (+2 to +7) with an intensity threshold set between 5.0 × 10^3^ and 1 × 10^20^ and a dynamic exclusion window of 35 s with ± 10 ppm. MS2 precursor ions were isolated in the quadrupole set to a mass width filter of 1.2 m/z. Ion trap fragmentation spectra (ITMS2) were collected with an AGC target setting of 1 × 10^4^, maximum injection time of 35 ms, and the CID collision energy set at 35%. This method takes advantage of multiple analyzers on Orbitrap Fusion Lumos, driving the system to use all available parallelizable time and decreasing the dependence on method parameters.

#### HyPro-FISH

Proximity-labeled samples on 18-mm coverslips prepared as described above were rinsed with 1 × PBS and 4 × SSC and incubated with A647-conjugated streptavidin (Biolegend, cat# 405237; 1:200 dilution) in HyPro blocking buffer with 80 units/ml murine RNase inhibitor at room temperature for 1h. The cells were washed with 4 × SSC, 4 × SSC and 0.1% Triton X-100, and 4 × SSC, 10 min each wash, briefly rinsed in 1 × PBS, stained with 0.5 μg/ml 4′,6-diamidino-2-phenylindole (DAPI) in 1 × PBS for 3 min at room temperature, and mounted onto microscope slides using ProLong Gold antifade reagent (Thermo Fisher Scientific, cat# P36934). Images were taken using ZEISS Axio Observer Z1 Inverted Microscope with alpha Plan-Apochromat 100 × /1.46 oil immersion objective. Z stacks were taken at 0.22-μm intervals.

#### Immunofluorescence and RNA-FISH

Cells grown on 18-mm coverslips were rinsed once with 1 × PBS. We used two alternative fixation/permeabilization strategies depending on the antibody. For antibodies against fibrillarin (Abcam, cat# ab5821; RRID:AB_2105785; 1:500 dilution), hnRNPL (Abcam, cat# ab32680; RRID:AB_941986; 1:500 dilution) or MCM5 (Abcam, cat# ab75975; RRID:AB_1310439; 1:50 dilution), cells were incubated in CSK buffer (100 mM NaCl, 300 mM sucrose, 10 mM PIPES, pH 6.8, 3 mM MgCl_2_) with 0.5% Triton X-100 and 80 units/ml murine RNase inhibitor for 4 min on ice, fixed with 4% formaldehyde diluted in 1 × PBS (ThermoFisher Scientific, cat# 28908) for 15 min at room temperature, and washed 3 times with 1 × PBS at room temperature, 5 min each wash. For antibodies against SFPQ (Abcam, cat# 38148; RRID:AB_945424; 1:200 dilution) and PTBP1 (Abcam, cat# ab133734; RRID:AB_2814646; 1:200), cells were fixed with 4% formaldehyde (ThermoFisher Scientific) for 15 minutes at room temperature, washed three times with 1 × PBS for 5 minutes each, permeabilized with 70% ethanol for 1 hour at room temperature or 4°C overnight and rinsed with 1 × PBS.

In either case, cells were blocked in the IF-blocking buffer (1% BSA and 0.2% Tween 20 in 1 × PBS) for 30 min at room temperature, and incubated with an appropriate antibody in the IF-blocking buffer additionally containing 20 units/ml murine RNase inhibitor for 1 hour at room temperature or overnight at 4°C. The coverslips were then washed three times with 1 × PBS, and incubated with corresponding Alexa Fluor-conjugated secondary antibodies for 1 hour at room temperature, followed by three washes with 1 × PBS, fixing the signal with 4% formaldehyde for 15 min at room temperature, and another three washes with 1 × PBS.

The subsequent RNA-FISH staining steps were performed as follows. Digoxigenin-labeled oligo probes prepared as described above were diluted in hybridization buffer (2 × SSC, 10% formamide and 10% dextran sulfate) and incubated with the coverslips overnight at 37°C. The samples were then washed with 2 × SSC and 10% formamide at 37°C for 30 min and 1 × SSC at room temperature for 15 min, blocked with 1 × PBS, 3% BSA and 100 units/ml murine RNase inhibitor at room temperature for 30 min, and incubated with mouse anti-digoxigenin antibody (Jackson Laboratories, cat# 200-002-156; RRID:AB_2339005; 1:500 dilution) for overnight at 4°C This was followed by washes in 4 × SSC, 4 × SSC and 0.1% Triton X-100, and 4 × SSC, 10 min each, and incubation with Alexa Fluor 647-anti-mouse secondary antibody in IF-blocking buffer (ThermoFisher Scientific, cat# A31571; RRID:AB_162542; 1:300 dilution) for 1 hour at room temperature. The samples were finally washed with 4 × SSC, 4 × SSC and 0.1% Triton X-100, and 4 × SSC, 10 min each, briefly rinsed in 1 × PBS, stained with 0.5 μg/ml DAPI in 1 × PBS for 3 min at room temperature, and mounted onto microscope slides using ProLong Gold antifade reagent (Thermo Fisher Scientific, cat# P36934). Images were taken using a ZEISS Axio Observer Z1 Inverted Microscope with alpha Plan-Apochromat 100x/1.46 oil immersion objective. Z stacks were taken at 0.22-μm intervals.

#### Combined RNA- and DNA-FISH

HeLa cells on 18-mm coverslips were incubated in CSK buffer with 0.5% Triton X-100 and 80 units/ml murine RNase inhibitor for 4 min on ice, fixed with 4% formaldehyde in 1 × PBS for 15 min at room temperature, and washed 3 times with 1 × PBS, 5 min each wash. RNA-FISH staining was then carried out as described in the “Immunofluorescence and RNA-FISH” section. Following the secondary antibody incubation and washing steps, cells were post-fixed with 4% formaldehyde in 1 × PBS for 15 minutes at room temperature, washed three times with 1 × PBS and treated with 100 μg/ml RNase A (Thermo Fisher Scientific, cat# EN0531) for 1 hour at 37°C and washed three times with 1 × PBS. The coverslips were then incubated at room temperature in 1 × PBS with 0.2 M HCl and 0.5% Tween-20 for 10 min, washed twice with 1 × PBS and 0.2% Tween-20, 3 min each wash, and transferred to 2 × SSC. Genomic DNA was then denatured by incubating the coverslips in 70% formamide, 2 × SSC at 80°C for 5 min. The samples were dehydrated with increasing concentrations of ice-cold ethanol (70%, 90% and 100%; 2 min each change) and air-dried at 42°C. Denatured nick-translated BAC probes prepared as described above were then added directly to the dry samples and incubated overnight at 37°C in a humidified chamber. On the next day, samples were washed with 50% formamide, 2 × SSC for 15 min at 37°C, followed by 2 × SSC for 15 min at 37°C, and 1 × SSC for 15 min at room temperature. Cells were stained with 0.5 μg/ml DAPI in 1 × PBS for 3 min at room temperature, mounted onto microscope slides using ProLong Gold antifade reagent (Thermo Fisher Scientific, cat# P36934), and imaged as described above.

#### Analyses of high-throughput sequencing data

Human genome sequence (GRCh38.primary_assembly.genome.fa.gz) was downloaded from (https://www.gencodegenes.org/human/release_32.html). To prepare an extended transcriptome file (extended.gtf), we first supplemented gencode.v32.basic.annotation.gtf.gz from https://www.gencodegenes.org/human/release_32.html with RefSeq 45S (NR_146144.1, NR_146151.1 and NR_146117.1), 28S (NR_146148.1, NR_146154.1 and NR_146118.1) and 18S (NR_146146.1, NR_146152.1 and NR_146119.1) ribosomal RNA entries mapping to a single canonical chromosomal position and maximally one non-assembled contig in GRCh38. We then appended the resultant gencode_rRNA.gtf file with high-confidence lncRNA entries from LNCpedia (https://lncipedia.org/downloads/lncipedia_5_2/high-confidence-set/lncipedia_5_2_hc_hg38.bed) that were > 20% distinct from GENCODE lncRNA annotations. These were shortlisted using BEDTools (Quinlan and Hall, 2010):

bedtools intersect -v -s -split -f 0.8 -a lncipedia_5_2_hc_hg38.bed12 ∖
b gencode_rRNA.bed6 > lncipedia_distinct.bed12

The gencode_rRNA.gtf and lncipedia_different.bed12 files were consolidated in gencode_rRNA_lncipedia.gtf and used as a reference to mine HyPro-seq data for novel transcripts using the HISAT2-StringTie pipeline (Pertea et al., 2016). For this purpose, known splice sites (gencode_rRNA_lncipedia.ss.txt) were extracted using the hisat2_extract_splice_sites.py script packaged with HISAT2. Quality of HyPro-seq FASTQ files (original.fastq) was inspected by FASTQC (https://www.bioinformatics.babraham.ac.uk/projects/fastqc/) and the reads were quality-trimmed by Trimmomatic (Bolger et al., 2014):

java -jar trimmomatic-0.38.jar SE -phred33 -threads [n_threads] [original.fastq] ∖
[trim.fastq] LEADING:10 TRAILING:10 SLIDINGWINDOW:5:20 MINLEN:38

Trimmed reads for the four technical replicates for each HyPro-seq experiment (trim1.fastq, trim2.fastq, trim3.fastq and trim4.fastq) were then aligned as follows:

hisat2 -p [n_threads] -k 50 --rna-strandness R --dta-cufflinks --no-unal ∖
- -known-splicesite-infile gencode_rRNA_lncipedia.ss.txt ∖
x GRCh38.primary_assembly.genome.index ∖
U [trim1.fastq],[trim2.fastq],[trim3.fastq],[trim4.fastq] ∖
S [hisat.sam]

The resultant SAM files (hisat.sam) were converted to BAM format (hisat.bam) using SAMtools (Li et al., 2009) and sample-specific transcriptomes (transcript.gtf) were assembled as follows:

stringtie -p [n_threads] --rf -M 1 -u -j 3 -c 3 -s 3 -f 0.2 ∖
G gencode_rRNA_lncipedia.gtf ∖
o [transcript.gtf] [hisat.bam]

The transcriptome files were merged:

cuffmerge -p [n_threads] --min-isoform-fraction 0.1 ∖
g gencode_rRNA_lncipedia.gtf -o merged.gtf ∖
[list_of_gtf_files]

and novel intergenic transcripts likely corresponding to previously unannotated lncRNAs were shortlisted:

grep ‘class_code ∖”u∖”‘ merged.gtf > merged_novel.gtf

We finally concatenated gencode_rRNA_lncipedia.gtf with merged_novel.gtf and strRNA entries from our previous study (Yap et al., 2018) to produce the extended.gtf transcriptome annotation file.

Transcript abundance in quality-trimmed HyPro-seq samples was quantified by Kallisto (Bray et al., 2016) with an extended.gtf-based index:

kallisto quant -t [n_threads] --rf-stranded --single -l 250 -s 50 ∖
i extended.index -o [out_dir] [trim.fastq]

Transcript-specific HyPro-seq counts for individual biological and technical replicates were imported into R (R Core Team, 2019) using the tximport package (Soneson et al., 2015) and analyzed by DESeq2 (Love et al., 2014). Genes represented by < 5 sequencing reads in > 50% of samples were considered not detectably expressed and were excluded from the DESeq2 analyses. A subset of detectably expressed genes depleted from compartment-specific HyPro-seq samples (< 1-fold enrichment versus HyPro-infusion control) or failing to show significant enrichment (< 1.5-fold enrichment, FDR > 0.1) were classified as HyPro-unlabeled. The above Kallisto-tximport-DESeq2 pipeline was also used to analyze APEX-seq (Fazal et al., 2019) (https://www.ncbi.nlm.nih.gov/geo/query/acc.cgi?acc=GSE116008; samples GSM3206948, GSM3206949, GSM3206950 and GSM3206951) and RNA-seq data(Wang et al., 2018) (https://www.ncbi.nlm.nih.gov/geo/query/acc.cgi?acc=GSE110775; samples GSM3016459, GSM3016460, GSM3016461).

Chromosome regions containing larger than expected numbers of HyPro-seq targets were identified by sliding a 5-Mb window with a 2.5-Mb step and comparing the real incidence of targets with that expected by chance using one-sided Fisher’s exact test. The probability of gene clustering by chance was further estimated by comparing median intergenic distances of real HyPro-seq targets with those calculated for simulated data as described (Thévenin et al., 2014). Random sampling was done in R using the following function:

sample(coord[i], n_per_chr[i], replace = FALSE)

where [i] was selected from canonical chromosomes containing > 1 HyPro-seq gene; coord[i] is a vector of midpoint coordinates of all detectably expressed genes on [i]; and n_per_chr[i] is the number of HyPro-seq genes per [i].

After calculating simulated intergenic distances for all qualifying chromosomes, the real and simulated distance medians were compared. This was repeated n=10,000 times and the P value for discovering gene clustering by chance was calculated as follows:

$$P\;=\;\frac{k+1}{n+\;1}$$

where k is the number of times where simulated median is smaller than the real median.

HeLa NAD regions were downloaded from (Németh et al., 2010); CHART-seq peaks, from (West et al., 2014); and the RepeatMasker track containing Alu and MIR SINEs, from http://genome.ucsc.edu/cgi-bin/hgTables. We extracted HeLa hg19 Hi-C data (Rao et al., 2014) for the *NEAT1*-comprising region using the Python module hic-straw (https://github.com/aidenlab/straw/wiki/Python):

neat1_hic_chr[i] = straw.straw("KR", "https://hicfiles.s3.amazonaws.com/hiseq/hela/in−situ/combined.hic", "11:65000000:65500000", "[i]", "BP", 500000)

where [i] was selected from chromosomes 1, ..., 22, X.

hic-straw was also used to extract NEAT1-RNA interaction events from RIC-seq (Cai et al., 2020) rRNA-depleted hg19 HIC files (https://www.ncbi.nlm.nih.gov/geo/query/acc.cgi?acc=GSE127188; samples GSM3629915 and GSM3629916):

neat1_ric_chr[i] = straw.straw("NONE", "[RICseq.hic]", "11:65190000:65212999", "[i]", "BP", 1000)

where [i] was selected from chromosomes 1, ..., 22, X.

Transcripts produced from specific genes were considered detectably crosslinked to NEAT1 RNA only when they were identified in both GSM3629915 and GSM3629916 replicates.

To deduce transcript abundance from RIC-seq sequencing data, we first removed adaptor sequences from the original GSM3629915 and GSM3629916 paired-end FASTQ files using cutadapt (Martin, 2011):

cutadapt -j [n_threads] -m 38 --error-rate = 0.05 --match-read-wildcards ∖
a AGATCGGAAGAGCACACGTCTGAACTCCAGTCA ∖
A AGATCGGAAGAGCGTCGTGTAGGGAAAGAGTGT ∖
o [cutadapt1.fastq] -p [cutadapt2.fastq] ∖
[original1.fastq] [original2.fastq]

and then quality-trimmed the reads as follows:

java -jar trimmomatic-0.38.jar PE -phred33 -threads [n_threads] ∖
[cutadapt1.fastq] [cutadapt2.fastq] ∖
[trim1.fastq] [trim1.unpaired.fastq] [trim2.fastq] [trim2.unpaired.fastq] ∖
LEADING:10 TRAILING:10 SLIDINGWINDOW:5:20 MINLEN:38

Non-hybrid, non-spliced reads were shortlisted using HISAT2 and SAMtools:

hisat2 -p [n_threads] -k 50 --fr --no-spliced-alignment --no-mixed --no-discordant ∖
X 500 --no-unal -x GRCh38.primary_assembly.genome.index ∖
−1 [trim1.fastq] −2 [trim2.fastq] -S [nonhybrid.sam]
samtools fastq -n −1 [nonhybrid1.fastq] −2 [nonhybrid1.fastq] [nonhybrid.sam]

The resultant FASTQ files were processed in Kallisto:

kallisto quant -t [n_threads] --rf-stranded -i extended.index ∖
o [out_dir] [nonhybrid1.fastq] [nonhybrid2.fastq]

and gene-specific expression estimates (in tpm) were extracted by tximport.

To estimate gene coverage by transcribing Pol II, HeLa mNET-seq (Nojima et al., 2015) paired-end FASTQ files were downloaded from https://www.ncbi.nlm.nih.gov/geo/query/acc.cgi?acc=GSM2357382, and processed by cutadapt:

cutadapt -j [n_threads] -a TGGAATTCTCGGGTGCCAAGG ∖
A GATCGTCGGACTGTAGAACTCTGAAC --minimum-length 25 ∖
o [cut_mnetseq1.fastq] -p [cut_mnetseq2.fastq] ∖
[original_mnetseq1.fastq] [original_mnetseq2.fastq]

The data were then pseudoaligned using Kallisto and counts per million per kb (cpmk) were calculated in R using tximport. We also used R to select a subset of NEAT1 HyPro-unlabeled genes with the highest Pol II cpmk values, such that the distribution median matches that of NEAT1 HyPro-labeled genes (e.g., Figures 6B and 6C).

To identify high-quality A-to-I RNA editing events in HISAT2-aligned NEAT1 HyPro-seq data, we first marked possible duplicates using SAMtools and Picard (http://broadinstitute.github.io/picard/):

samtools calmd -@ [n_threads] --output-fmt BAM ∖
[hisat.bam] GRCh38.primary_assembly.genome.fa > [hisat.MD.bam]
samtools index -@ [n_threads] [hisat.MD.bam]
java -jar picard.jar MarkDuplicates ∖
I = [hisat.MD.bam] ∖
O = [marked_duplicates.bam] ∖
M = [marked_dup_metrics.txt]

Modified nucleotide positions were then identified using JACUSA (Piechotta et al., 2017):

java -jar JACUSA_v1.3.0.jar call-1 -p [n_threads] ∖
F 1024 -f B -P RF-FIRSTSTRAND ∖
r [jacusa.bed] [marked_duplicates.bam]

The resultant [jacusa.bed] file containing putative RNA editing events was filtered from known single-nucleotide polymorphisms from ftp://ftp.ncbi.nlm.nih.gov/snp/organisms/human_9606_b151_GRCh38p7/VCF/GATK/All_20180418.vcf.gz using bedtools intersect -v. Strand-specific A-to-I editing events detectable as A-to-G changes in sequencing data were shortlisted in R and summarized for inverted Alu repeats as a fraction of total coverage (Figures 6E–6G; Figures S6D–S6F).

We used IGV (Robinson et al., 2011) to visualize rpm-normalized strand-specific bedGraph coverage files produced from HISAT2-derived BAM files prepared as follows:

bedtools genomecov -ibam [hisat.bam] -bg -scale [scale_factor] -split ∖
strand + > [plus. bedGraph]
bedtools genomecov -ibam [hisat.bam] -bg -scale [scale_factor] -split ∖
strand - > [minus. bedGraph]

where [scale_factor] was calculated as 1000000/[total_n_aligned_reads].

Genomic coordinates were converted between GRCh38/hg38 and earlier genomic assemblies using the https://genome.ucsc.edu/cgi-bin/hgLiftOver tool. Different sets of genomic regions were intersected using BEDTools (Quinlan and Hall, 2010). To prepare the heatmap in Figure 3A, VST-transformed (Love et al., 2014) expression values of the 50 most variable genes enriched in compartment-specific HyPro-seq data with FDR < 1E-4 were processed in pheatmap (https://cran.r-project.org/web/packages/pheatmap/). Distributions of HyPro-seq labeled transcripts and other features along the chromosomes were visualized in karyoploteR (Gel and Serra, 2017). HyPro-seq coverage metaplots were prepared using ngs.plot (Shen et al., 2014). Intron retention was analyzed using IRFinder and the Audic and Claverie test (Middleton et al., 2017).

#### Mass spectrometry data analyses

Raw mass-spec data files were processed using Proteome Discoverer (v2.2; Thermo Fisher Scientific, UK) to search against Uniprot Swissprot *Homo sapiens* Taxonomy (49,974 entries) using Mascot (v2.6.0; www.matrixscience.com) and the Sequest search algorithms (Eng et al., 1994). Precursor mass tolerance was set to 20 ppm with fragment mass tolerance set to 0.8 Da with a maximum of two missed cleavages. Variable modifications included carbamidomethylation (Cys) and oxidation (Met). Searching stringency was set to 1% False Discovery Rate (FDR). In total, 2213 proteins were detected in our HyPro-MS data. Normalized total spectra quantitative values for individual proteins were estimated in Scaffold (v 4.11.0; https://www.proteomesoftware.com). The quantitative value data were imported into R as proteins per million (fraction of the total multiplied by 1E6) and analyzed using the DEP package (Zhang et al., 2018) (https://bioconductor.org/packages/release/bioc/vignettes/DEP/inst/doc/DEP.html). We filtered the data to include proteins identified in all 3 replicates of at least one condition (either compartment-specific or control HyPro-MS) and used default imputation settings (fun = “MinProb,” q = 0.01). DEP-generated *P*-values were adjusted for multiple testing using the Benjamini-Hochberg (FDR) method. Lists of proteins localizing to the nucleolus or/and nucleus as their “Main.location” were downloaded from the Human Protein Atlas (https://www.proteinatlas.org/about/download) and filtered to remove “Uncertain” reliability entries. The list of high-confidence paraspeckle markers was from (Naganuma et al., 2012). Proteins enriched by NEAT1 CHART-MS were shortlisted from Table S6 in (West et al., 2014) by requiring that their abundance in both capture oligonucleotide experiments (CO1 and CO2) exceed input levels > 2-fold, both input-normalized CO1 and CO2 signals are stronger compared to the sense oligonucleotide control, and the signal in at least one CO experiment exceeds its sense counterpart > 2-fold.

Specificity index (*SI*) was calculated for protein j and compartment k∈{1,...,n} in principle as in (Julien et al., 2012):

$$SI_{j,k}\;=\;\frac{LFE_{j,k}}{\sum_{i=1}^{n}LFE_{j,i}}$$

Where LFE is non-negative log_2_-transformed fold enrichment versus the HyPro-infusion control, and n is the number of compartments (n=3 in our case). Non-positive log_2_-transformed fold enrichment values were set to 0.

Gene ontology and MCODE protein network analyses were carried out using Metascape (Zhou et al., 2019b) (https://metascape.org).

#### Microscopy data analyses

Interphase nuclei were identified by DAPI staining and used for subsequent analyses. HyPro-seq target-specific weighted Manders’ coefficients for colocalization with RNA baits were calculated using the Colocalization module of ZEN Blue software (ZEISS). Voxel-integrated densities of RNA-FISH signals and distances between distinct RNA-FISH objects (border-to-border) or between DNA-FISH signals and RNA-FISH objects (centroid-to-border) were analyzed using the 3D ImageJ suite (Ollion et al., 2013) (https://imagej.net/3d-imagej-suite).

### Quantification and statistical analysis

All statistical procedures were performed in R. Unless stated otherwise bioinformatics and imaging data were compared by two-tailed Wilcoxon rank sum test, Fisher’s exact or χ^2^ test, as appropriate. Data obtained from RT-qPCR were analyzed using a two-tailed paired Student’s t test. Correlation was analyzed using the Pearson’s product-moment method. Where necessary, *P*-values were adjusted for multiple testing using the Benjamini-Hochberg (FDR) method. Numbers of experimental replicates, *P*-values and the tests used are indicated in the Figures and/or Figure legends.

### Additional resources

#### Detailed protocol

A step-by-step description of HyPro labeling procedures can be found in Methods S1.

## Data Availability

• The HyPro-seq data have been deposited to ArrayExpress and the HyPro-MS data, to the ProteomeXchange Consortium via the PRIDE (Perez-Riverol et al., 2019) partner repository. All data are publicly available as of the date of publication with the accession numbers listed in the key resources table. • This paper does not report original code. Routine bioinformatics approaches used to analyze the data are described in relevant STAR Methods sections. • Any additional information required to reanalyze the data reported in this paper is available from the lead contact upon request.

## Supplemental information

Document S1. Figures S1–S7 and Methods S1

Table S1. Probe sets used for HyPro-FISH and RNA-FISH, related to STAR Methods

Table S2. Proteins specifically labeled in HyPro-MS experiments, related to Figure 2

Table S3. Significantly enriched HyPro-seq targets, related to Figures 3 and 4

Table S4. NEAT1 HyPro-seq targets (pink) and unlabeled controls (gray) selected for experimental validation, related to Figures 4 and 5

Table S5. Comparison of NEAT1 HyPro-seq targets from chromosomes over-represented in ARPE-19 and HeLa, related to Figure 7

Document S2. Article plus supplemental information
