# Supplementary material for: Hybridization-proximity labeling reveals spatially ordered interactions of nuclear RNA compartments
Source: Mol Cell. 2022 Jan 20;82(2):463–478.e11. doi: 10.1016/j.molcel.2021.10.009 (PMC8791277; doi:10.1016/j.molcel.2021.10.009)
Supplement: Document S1. Figures S1–S7 and Methods S1 [file mmc1.pdf]

**Molecular Cell, Volume *82***

## Supplemental information

# Hybridization-proximity labeling reveals spatially ordered interactions of nuclear RNA compartments

**Karen Yap, Tek Hong Chung, and Eugene V. Makeyev**

# Supplemental Information

---

## Hybridization-proximity labeling reveals spatially ordered interactions of nuclear RNA compartments

Karen Yap, Tek Hong Chung and Eugene V. Makeyev

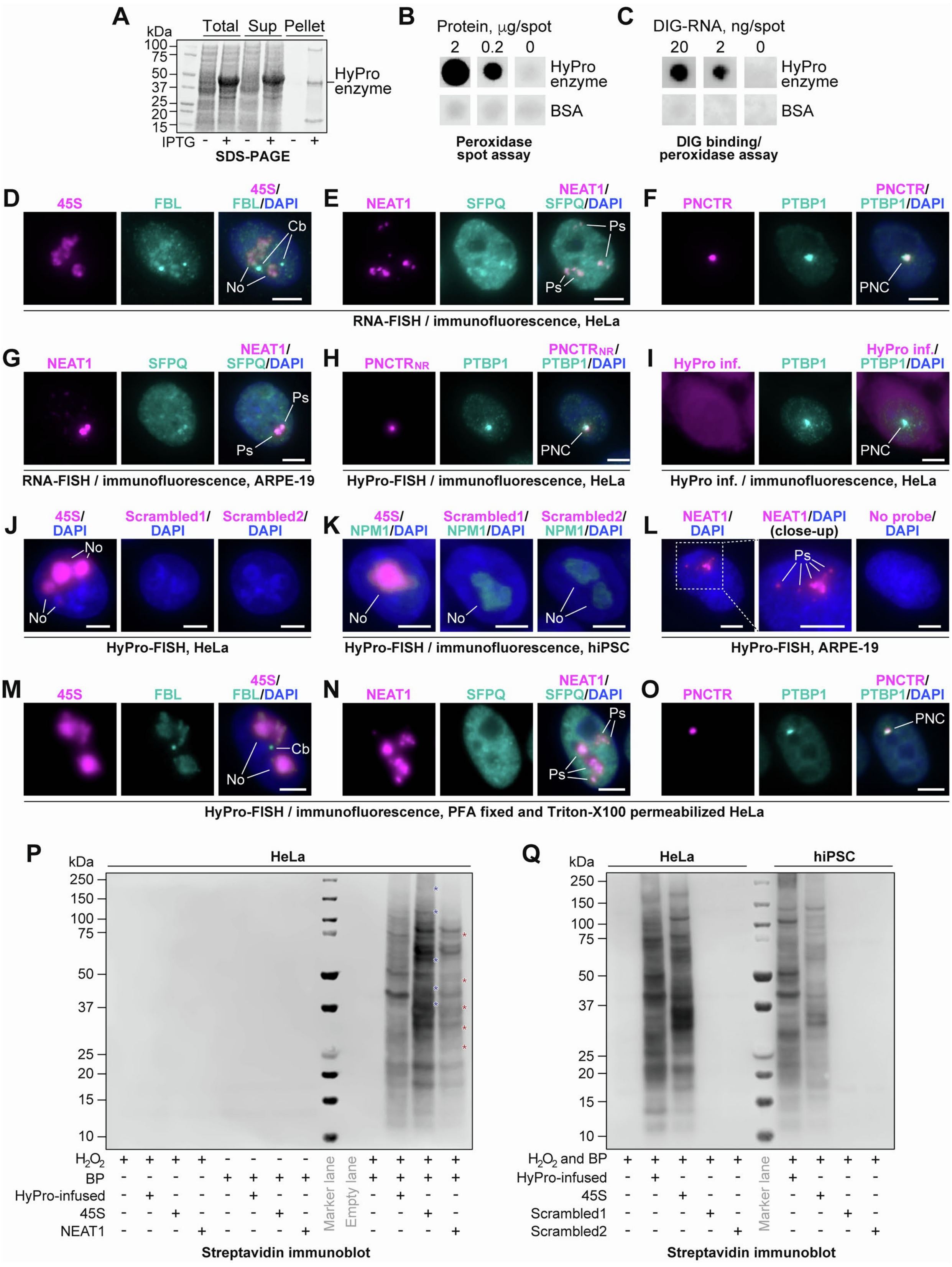


**Figure S1.** Development of the hybridization-proximity labeling technology. (Related to Figure 1).

(**A**) Inducible expression of soluble HyPro protein in recombinant *E. coli* cells. The bacteria were incubated for 24 hours at 25°C with and without IPTG, and total cellular proteins and the supernatant (Sup) and pellet fractions were analyzed by SDS-PAGE/Coomassie R-250 staining.

(**B**) Spot assay of peroxidase activity of purified HyPro.
(**C**) Another spot assay demonstrating that HyPro binds membrane-immobilized digoxigenin-labeled RNA retaining its peroxidase activity.
(**D-F**) Combined RNA-FISH/immunofluorescence analyses of HeLa cells with (D) 45S, (E) NEAT1 and (F) (UC)n-specific PNCTR probes showing that nucleoli (No), Cajal bodies (Cb), paraspeckles (Ps), perinucleolar compartment (PNC), and overall nuclear morphology are well preserved using the DSP crosslinking/ethanol permeabilization protocol optimized for in situ proximity labeling. FBL, a marker of No and Cb; SFPQ, a Ps marker also present in the nucleoplasm; PTBP1, a marker enriched in the PNC.
(**G**) RNA-FISH/immunofluorescence analysis carried out for NEAT1/paraspeckles in DSP/ethanol-treated non-transformed epithelial cell line ARPE-19.
(**H**) $PNCTR_{NR}$ probe set against non-repetitious PNCTR sequences labels the PNC in HyPro-FISH stained HeLa cells, similar to the probe targeting (UC)n-repeats (Fig. 1H).
(**I**) HyPro-FISH control where HeLa cells are incubated without a probe and then infused with with diluted HyPro enzyme for 5 min before the biotinylation step (HyPro inf.) results in homogeneous labeling of an entire cell.
(**J-K**) HyPro-FISH experiments showing that two distinct scrambled probe sets (Scrambled1 and Scrambled2) produce no detectable signal in (J) HeLa or (K) human induced pluripotent stem cells (hiPSC), while their non-scrambled 45S-specific counterpart stains nucleoli, as expected. Nucleolar outlines in (I) are visualized using an antibody against nucleophosmin (NPM1/B23).
(**L**) HyPro-FISH analysis of ARPE-19 cells. Note robust Ps staining in the sample hybridized with NEAT1-specific digoxigenin-labeled oligonucleotides (*left* panel and a close-up in the *middle*) but not in the negative control incubated without a probe (*right*).
(**M-O**) HyPro-FISH works well in HeLa cells fixed and permeabilized using the standard 4% formaldehyde / 0.1% Triton X-100 protocol. All scale bars in (D-O) are 5 μm.
(**P**) Fixed and permeabilized HeLa cells were HyPro-labeled with probes against 45S or NEAT1, incubated without a probe or incubated without a probe and infused with HyPro prior to the biotinylation reaction step (HyPro-infused). Biotinylated proteins were analyzed by immunoblotting with a streptavidin detection reagent. Note that efficient labeling depends on both proximity biotinylation substrates, i.e. biotin-phenol (BP) and $H_2O_2$, and requires HyPro to be retained in the sample either through interaction with digoxigenin (45S or NEAT1 lanes) or infusion. Also note that the two probe-specific samples differ from the HyPro-infused control and each other, with examples of lane-specific products marked by the asterisks.
(**Q**) Streptavidin immunoblot analysis of HeLa and hiPSC showing distinct protein labeling patterns in 45S probe and HyPro-infused samples and no detectable biotinylation in samples incubated with Scrambled1 or Scrambled2 probe sets.

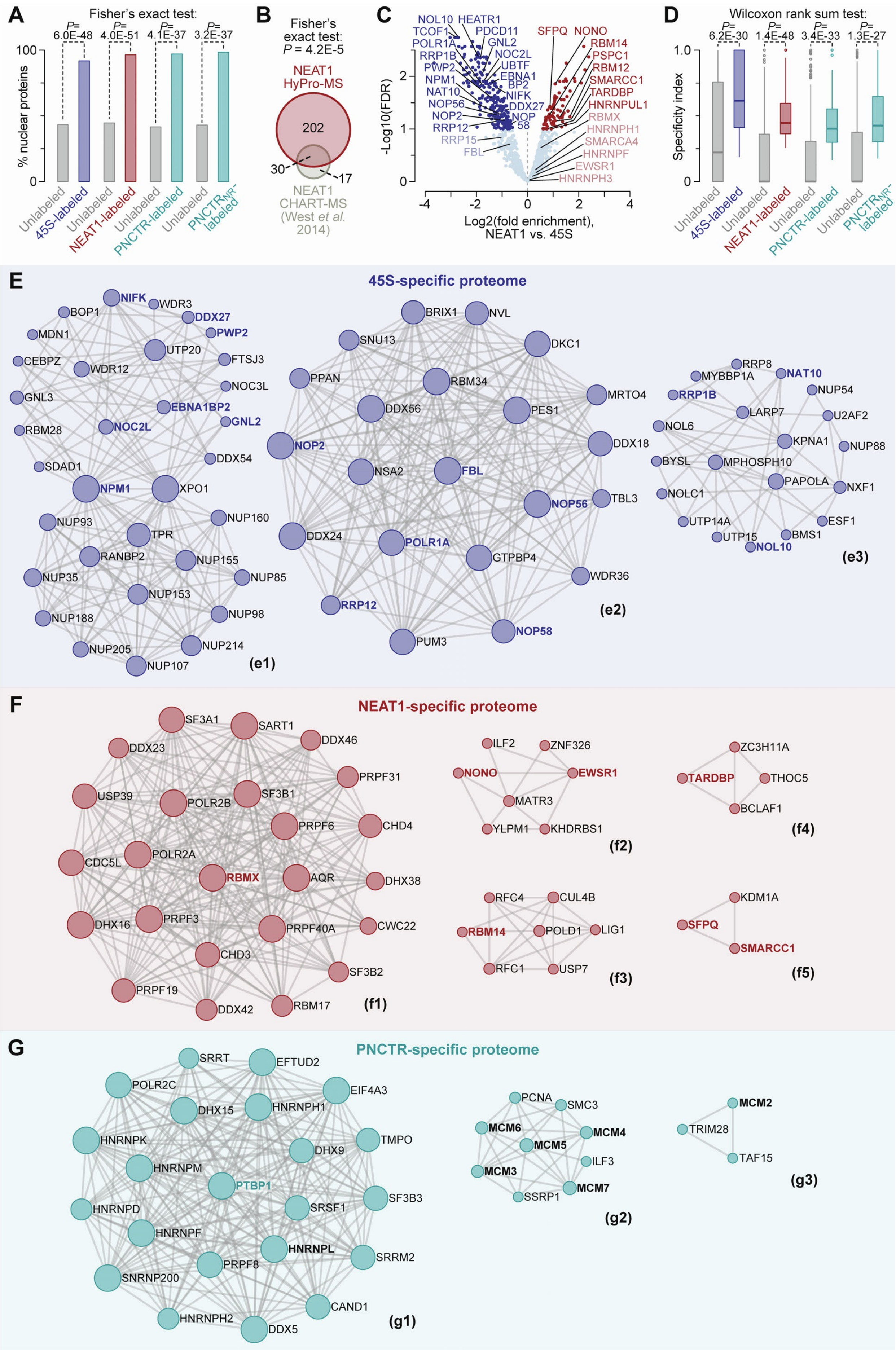


**Figure S2.** HyPro-MS identifies RNA compartment-specific proteomes. (Related to Figure 2).

(**A**) As expected, known nuclear proteins (https://www.proteinatlas.org/) are over-represented in the 45S-, NEAT1- and PNCTR-labeled sets (>2-fold, FDR<0.05) compared to detectably expressed but unlabeled controls.
(**B**) Proteins enriched in a NEAT1 CHART-MS study (West et al., 2014) are significantly over-represented in NEAT1 HyPro-MS-labeled proteome.
(**C**) Volcano plot comparison of 45S and NEAT1 HyPro-MS-labeled proteomes showing correct partitioning of nucleolar and paraspeckle markers from Fig. 2A-B to the corresponding quadrants. Proteins enriched in 45S or NEAT1 samples >1.5 fold with FDR<0.1 are shown as blue or red dots, respectively. Gray dots, the rest of proteins.
(**D**) HyPro-labeled proteomes are enriched for proteins specific to the corresponding RNA "bait". Data for the two PNCTR probes are analyzed separately in (A and D).
(**E-G**) Putative molecular complexes (MCODE clusters) extracted by Metascape (Zhou et al., 2019b) from (E) 45S-, (F) NEAT1- and (G) PNCTR-specific proteomes (Table S2). (E-F) Clusters e1-e3 and f1-f5 comprising known nucleolar (purple) and paraspeckle (red) markers introduced in Fig. 2A-B, respectively. (G) Cluster g1 comprising PNC marker PTBP1 (cyan) and two novel clusters, g2 and g3, containing subunits of the minichromosome maintenance complex (MCM). Proteins introduced in Fig. 2C are shown in bold.

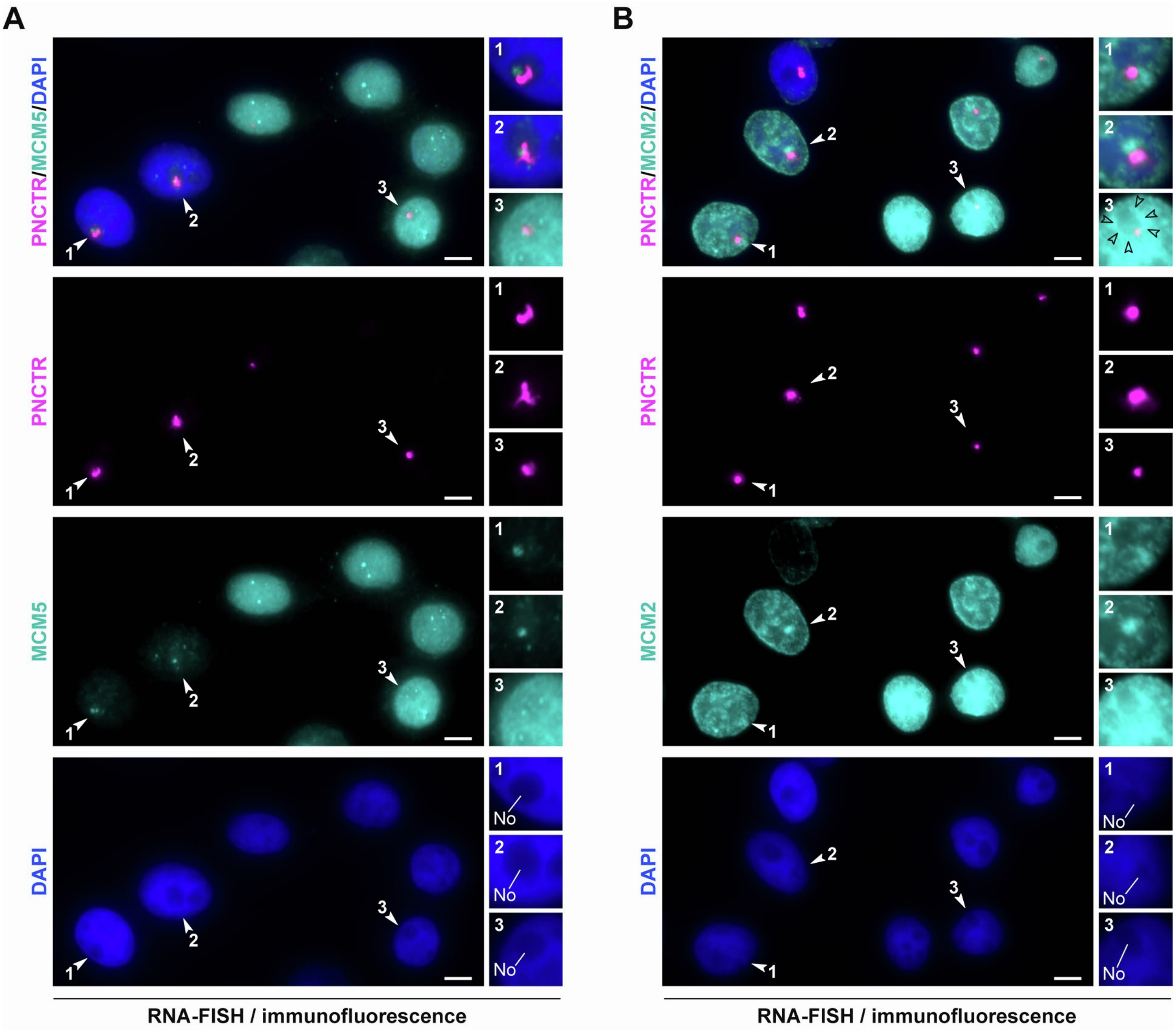


**Figure S3.** Recurrent clustering of MCM components near PNCTR-labeled compartments. (Related to Figure 2).

Combined RNA-FISH/immunofluorescence analyses show that PNCTR is often found near (**A**) MCM5- and (**B**) MCM2-containing perinucleolar structures. Note that the abundance of MCM5 and MCM2 proteins in the nucleoplasm differs depending on the cell, probably reflecting the cell cycle dependence reported earlier (Prasanth et al., 2004). No, nucleolus. Scale bars, 5 µm. White arrowheads in the main images indicate parts magnified 2-fold in the insets. Open arrowheads in the close-up 3 in (B) show extensive accumulation of MCM2 around nucleolus. The main images are maximum intensity Z-stacks and the close-ups are individual optical planes.

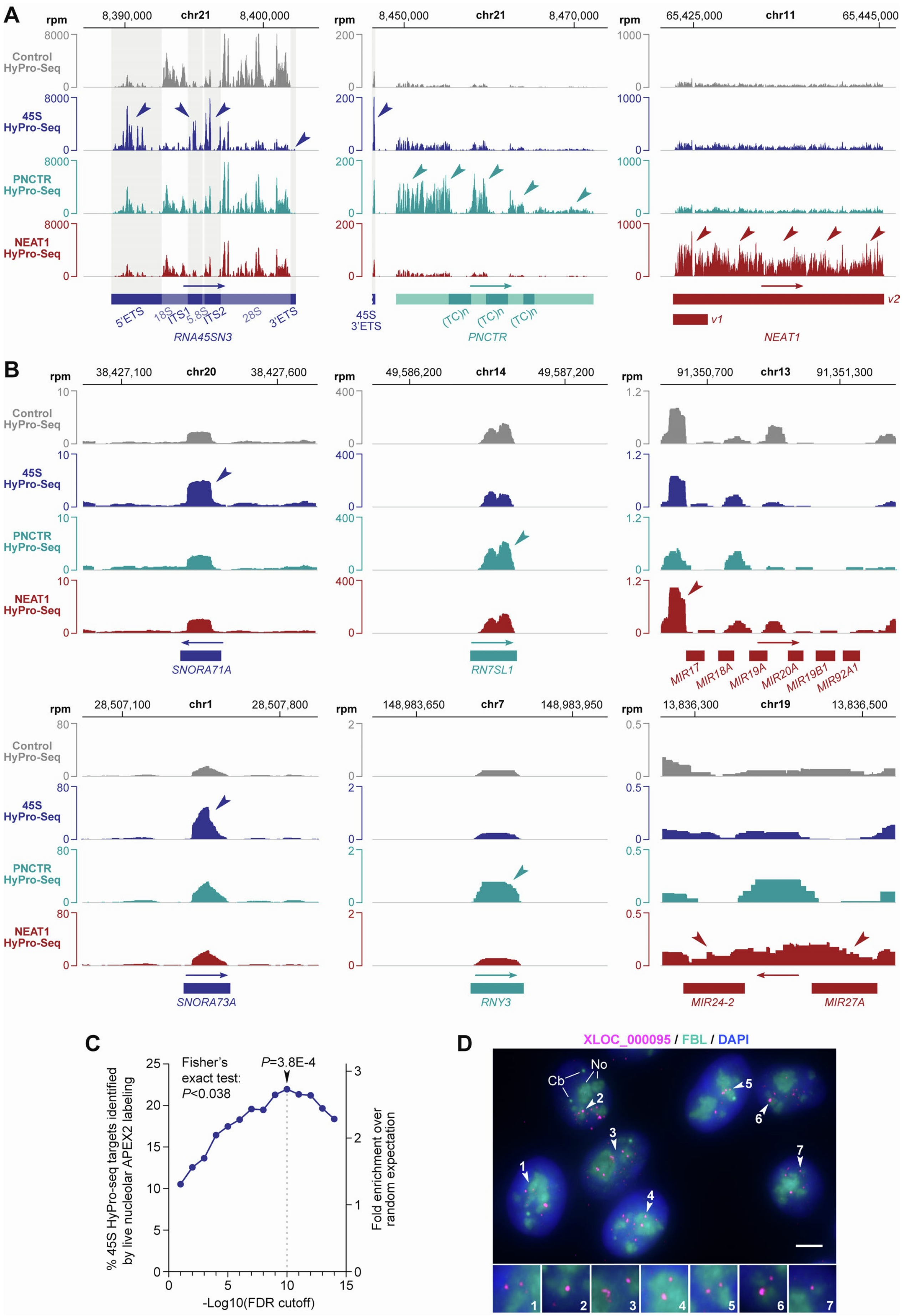


**Figure S4.** Initial validation of HyPro-seq data. (Related to Figure 3).

(**A**) Read per million (rpm) normalized coverage plots showing enrichment of the three RNA "baits" in the corresponding HyPro-seq datasets. Purple arrowheads, ETS and ITS spacer sequences present in the 45S pre-rRNA but not in mature rRNAs. Cyan arrowheads, enrichment of PNCTR sequences in PNCTR HyPro-seq. The apparently low coverage of the (TC)n sequences is likely due to the difficulty in mapping repeated sequences to their bona fide genomic origins. Red arrowheads, enrichment of the long (~23 kb) isoform of NEAT1 required for paraspeckle assembly (Naganuma et al., 2012) in NEAT1 HyPro-seq.
(**B**) Normalized coverage plots for RNA markers of nucleolus (left), the PNC (mid) and paraspeckles (right). The arrowheads mark sequences with increased coverage in corresponding HyPro-seq experiments.
(**C**) Overlap between transcripts labeled in vivo by nucleolar APEX2 (1.5-fold up, FDR<0.05) and 45S-proximal transcripts (1.5-fold up vs. the HyPro infusion control) plotted as a function of the HyPro-seq FDR. The overlap exceeds the number of transcripts expected by chance for all FDR values (Fisher's exact test $P<0.038$) and peaks at FDR=1E-10 with Fisher's $P$=3.8E-4.
(**D**) RNA-FISH/IF analysis confirming that the XLOC_000095 RNA identified by 45S HyPro-seq tends to localize near nucleoli (No; immunostained for the FBL protein). Scale bars, 5 µm. Arrowheads, areas magnified 2-fold in the close-ups at the bottom. Main images, Z-projections; close-ups, individual optical sections. Cb, Cajal bodies.

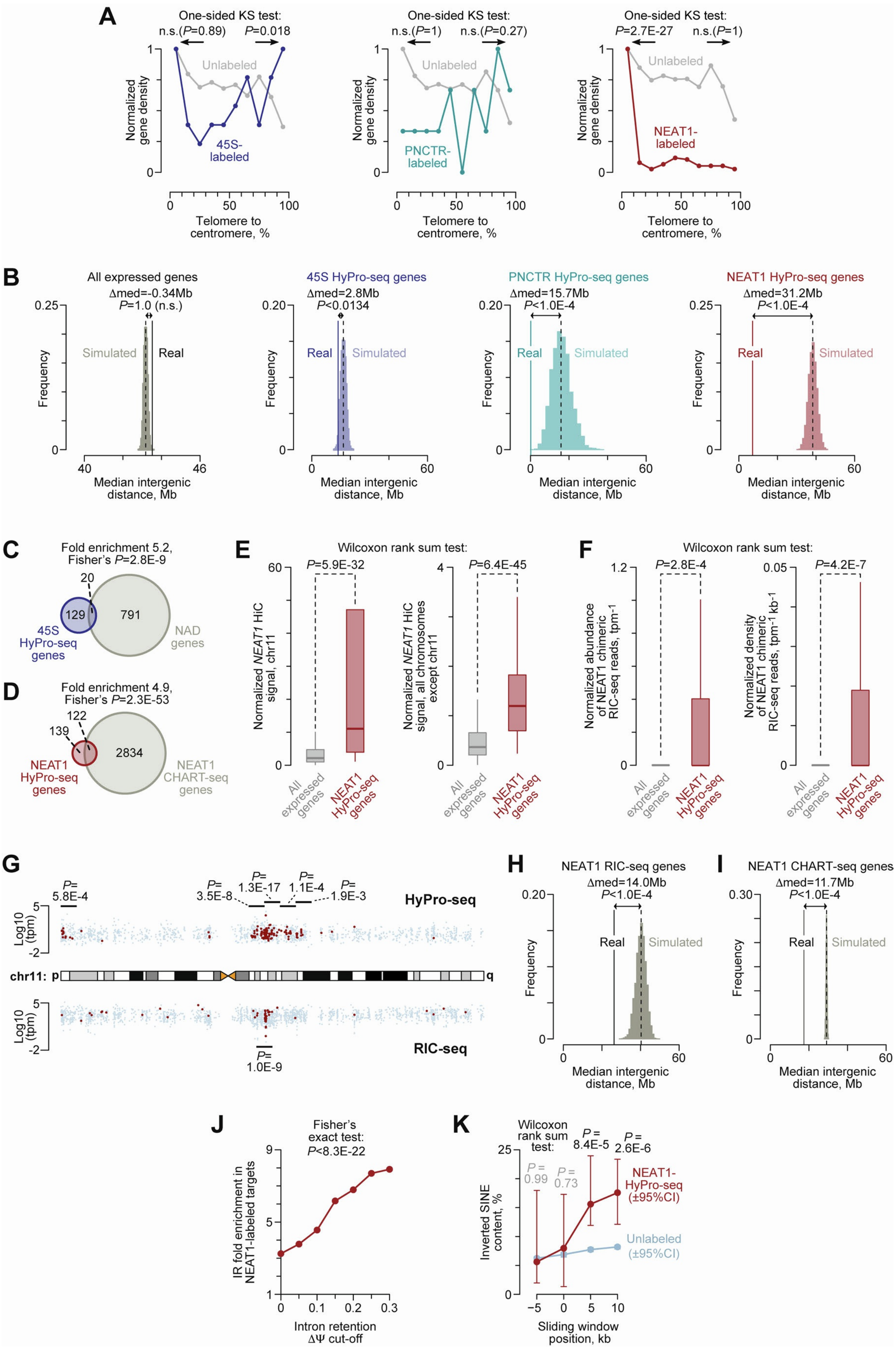


**Figure S5**. Bioinformatic characterization of HyPro-seq targets (Related to Figures 4 and 6). (**A**) Chromosomal position of genes encoding 45S-, PNCTR- and NEAT1-proximal transcripts

compared to all detectably expressed but unlabeled genes by one-sided KS test. Gene middle coordinates were placed into ten equally sized bins arranged in the telomere-to-centromere order for all HeLa chromosomes except those encoding the 45S, PNCTR or NEAT1 "baits" (i.e. chr11, chr13-15 and chr21-22). NEAT1 HyPro-seq genes are enriched in the 10% telomere-proximal bin, whereas 45S HyPro-seq genes tend to be located closer to the centromere.
(**B**) Comparisons between real (solid vertical lines) and simulated median intergenic distances (histograms) suggesting that genes encoding the 45S, PNCTR and NEAT1 HyPro-labeled transcripts cluster on chromosomes significantly tighter than expected by chance. NEAT1 HyPro-seq genes show especially large difference between the median of the simulated distribution (dashed vertical line) and the real median (Δmed = 31.2 Mb). This effect was not observed when the simulated distribution of all expressed genes by sampling from the entire pool of annotated genes (control graph on the left).
(**C**) 45S HyPro-seq genes are enriched in the NAD regions of the genome physically associated with the nucleolus (Nemeth et al., 2010).
(**D**) The overlap between genes encoding NEAT1-proximal transcripts and DNA sequences interacting with NEAT1 RNA in a CHART-seq experiment (West et al., 2014) is statistically significant.
(**E**) Quantification of the Hi-C data in (Fig. 4D) showing that NEAT1 HyPro-seq genes associate with the *NEAT1* locus more efficiently compared to all detectably expressed genes on both chr11 (*left*) and the rest of the chromosomes (*right*).
(**F**) A subset of NEAT1 HyPro-seq targets have relatively high abundance (i.e. number of normalized per transcript tmp expression value; *left*) and density (abundance per kb; *right*) of hybrid RIC-seq reads proximity-ligated reads with NEAT1 RNA (Cai et al., 2020).
(**G**) Unlike NEAT1 HyPro-seq that labels genetically distant clusters of genes, high-quality RIC-seq hits are concentrated in a narrow chr11q region immediately adjacent to the *NEAT1* locus, the only part of the genome significantly enriched in the sliding window analysis introduced in Fig. 4B-D. Black horizontal lines, non-overlapping 5-Mb sliding windows containing significantly larger than expected numbers of HyPro-seq or RIC-seq hits. Red, detectably expressed genes passing significance cutoffs; light blue, the rest of detectably expressed genes.
(**H-I**) Comparisons between real (solid vertical lines) and simulated median intergenic distances (histograms) suggesting that NEAT1-specific (H) RIC-seq (Cai et al., 2020) and (I) CHART-seq (West et al., 2014) hits are clustered on chromosomes tighter than expected by chance. Note, however, that the differences between real and simulated values in these comparisons (Δmed 11.7 and 14.0 Mb, respectively) are noticeably smaller than in the NEAT1 HyPro-seq analysis shown in (B).
(**J**) Fisher's exact test analysis showing that retained introns are progressively enriched in NEAT1 HyPro-labeled targets compared to the unlabeled, Pol II density-matched controls with increasing stringency of the ΔPSI cutoff.
(**K**) Inverted SINEs are more abundant in the 3' read-through region of NEAT1 HyPro-labeled targets compared to the unlabeled controls. Inverted repeat densities (minimum of forward and reverse densities multiplied by 2) in 10-kb windows centered at the positions indicated are plotted as medians ±95% confidence intervals and compared by two-tailed Wilcoxon test.

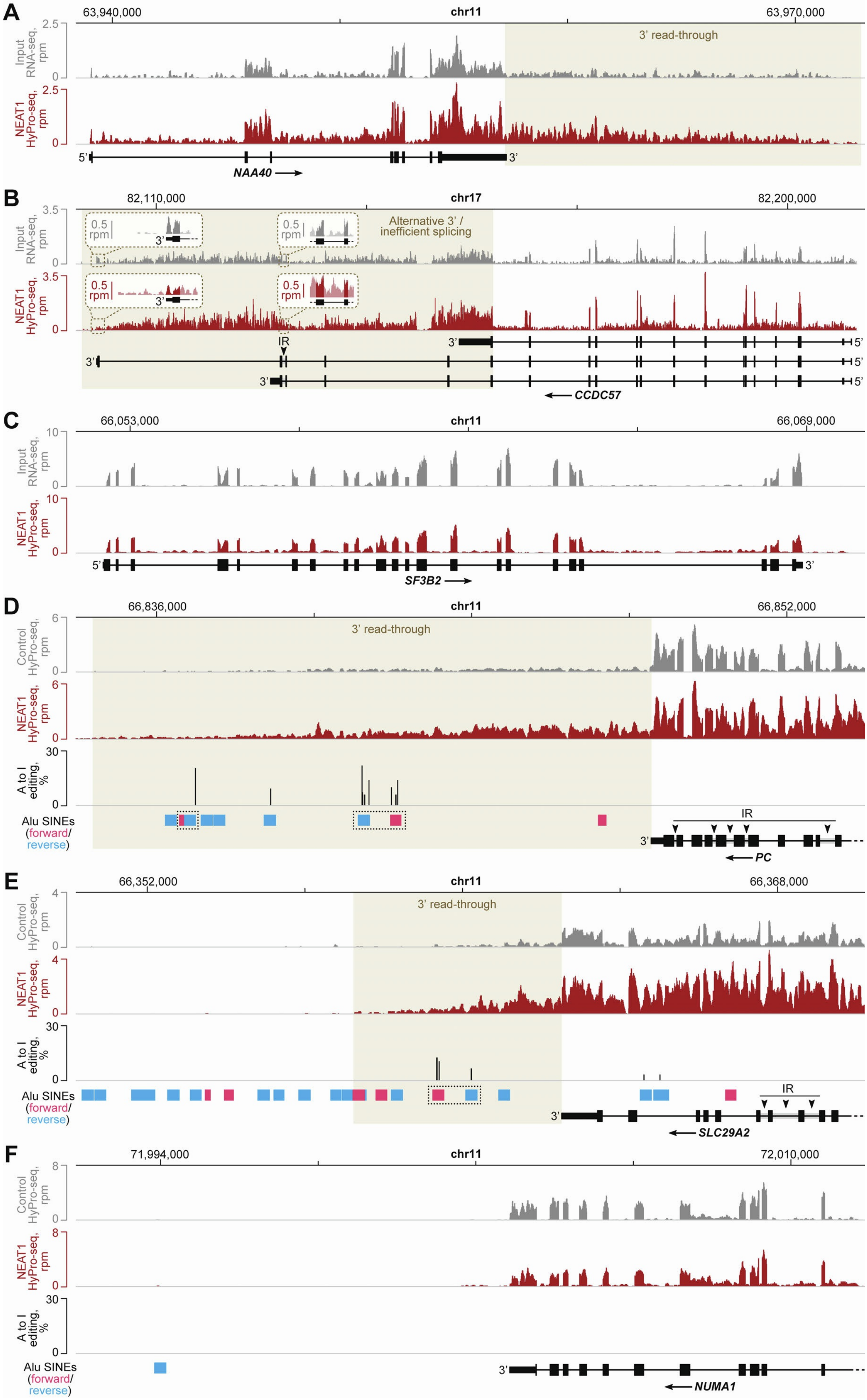


**Figure S6.** NEAT1 HyPro-seq targets are often poorly spliced and enriched in 3'-terminal

inverted Alu SINEs undergoing A-to-I editing. (Related to Figure 6).

(**A-C**) Comparison of NEAT1 HyPro-seq coverage plots (red) for NEAT-labeled targets (A) *NAA40* and (B) *CCDC57*, and (C) unlabeled control *SF3B2* with similarly normalized RNA-seq data for a total RNA fraction collected prior to the streptavidin capture step (gray). Similar to Fig. 6E-G, the Pol II read-through region of *NAA40* and the 3'-terminal part of *CCDC57* (including the significantly retained intron, IR) have better coverage in the NEAT1 HyPro-seq tracks compared to the total RNA. Close-ups in (B) show the IR- and the last exon-containing windows of *CCDC57* stretched horizontally by a factor of 5. Sequencing data for introns and the read-through region are rendered in lighter shades of gray and red, respectively.

(**D-F**) HyPro-infusion control and NEAT1 HyPro-seq coverage plots for NEAT proximity-labeled genes (D) *PC* and (E) *SLC29A2*, and (F) the unlabeled gene *NUMA1* (all from chr11q). Note extensive accumulation of NEAT1 HyPro-seq reads in the Pol II 3' read-through region and some introns of *PC* and *SLC29A2* but not *NUMA1*. Also note that the 3' read-through/3'-proximal regions of *PC* and *SLC29A2* but not *NUMA1* harbor inverted Alu SINEs undergoing A-to-I editing. Arrowheads, PC and SLC29A2 introns retained in NEAT1 HyPro-seq compared to the HyPro-infusion control with the >10% ΔPSI and <0.05 FDR IRFinder cutoffs. Dotted rectangles, inverted Alu units edited at more than one position.

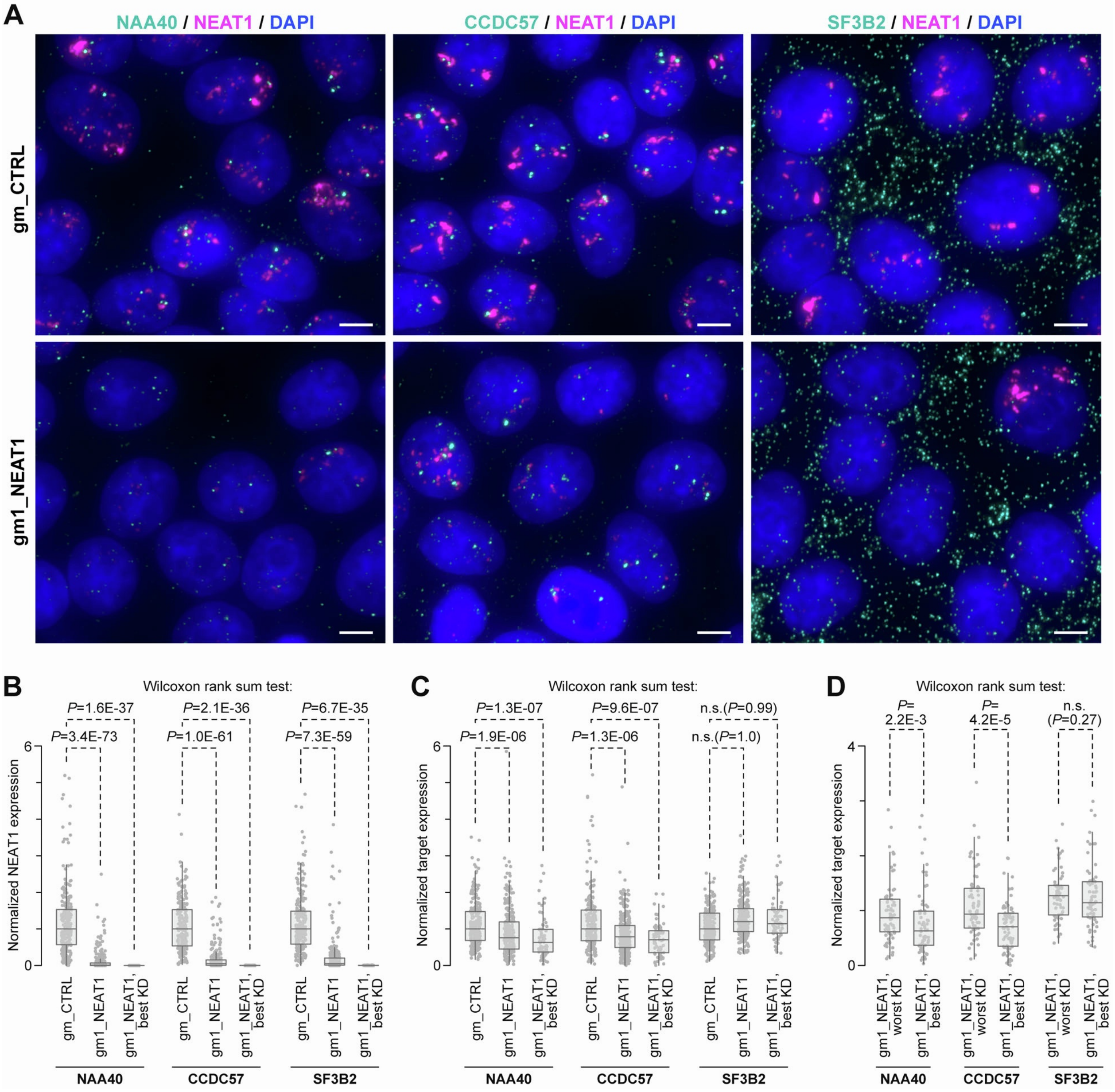

**Figure S7.** Optimal expression of paraspeckle-proximal targets depends on NEAT1. (Related to Figure 7).

(**A**) HeLa cells were transfected with either a non-targeting control (gm_CTRL; top row) or a NEAT1-specific gapmer (gm1_NEAT1; bottom row) and analyzed by two-color RNA-FISH. Note that the loss of NEAT1/paraspeckles in most gm1_NEAT1-treated cells correlates with visible reduction in NAA40 (left) and CCDC57 (mid) but not SF3B2 (right) signal intensity.

(**B-D**) Quantification of the data in (A). (B) Downregulation of nuclear NEAT1 RNA-FISH signals in an entire gm1_NEAT1 population and its lower quartile with the highest efficiency of NEAT1 knockdown (gm1_NEAT1, best KD). (C) Nuclear RNA-FISH signals of the NEAT1 HyPro-seq targets NAA40 and CCDC57 but not the negative control SF3B2 tend to decrease in response to gm1_NEAT1. This effect is especially evident in the cells with the lowest NEAT1 expression (gm1_NEAT1, best KD). (D) Comparison of nuclear RNA-FISH signals between the lower- (best KD; little to no NEAT1) and the upper-quartile cells (worst KD; relatively high residual NEAT1 levels) in gm1_NEAT1-treated samples. Note that the expression of NAA40 and CCDC57 but not SF3B2 correlates significantly with NEAT1 abundance. Expression values in (B-D) were normalized to the median of the corresponding gm_CTRL sample and compared by one-tailed Wilcoxon test. Total number of cells analyzed per sample, $n \geq 212$.

## Methods S1. Detailed HyPro labeling protocol. (Related to STAR*Methods).

### A. Solutions

***DSP (0.5 mg/ml)***

DSP is rather unstable in solution. Weigh out single-use aliquots and store them in microfuge tubes at -80°C in the powder form. Just before the cell fixation step, dissolve an aliquot in DMSO to make a 50 mg/ml stock solution. Add completely dissolved DSP to 1xPBS at room temperature dropwise with stirring to prepare the 0.5 mg/ml working solution. Do not use cloudy aqueous solutions of DSP or the fixation may not work properly.

***Biotin Phenol (Biotinyl tyramide, 500 mM)***

Prepare 500 mM stock by dissolving 100 mg biotin phenol in 550 µl DMSO. This may take a while, be patient. Store at -80°C in 10-50 µl single-use aliquots. Avoid repeated thawing and freezing.

***Hydrogen Peroxide (100 mM)***

Prepare 100 mM stock on the day of experiment by diluting 1 µl of commercial $H_2O_2$ solution (30% = ~10 M) in 99 µl of 1xPBS.

***Hybridization buffer (2xSSC, 10% formamide, 10% dextran sulfate)***

Dissolve 1 g of dextran sulfate in 1 ml 20xSSC, 1 ml 100% formamide topped up to 10 ml with nuclease-free water. This may take some time to dissolve completely. Store at 4°C for up to a month.

***Sodium Ascorbate (1 M)***

Prepare 1 M stock on the day of experiment by dissolving 0.198 g of sodium ascorbate in 1 ml of nuclease-free water.

***Trolox (500 mM)***

Prepare 500 mM stock on the day of experiment by dissolving 0.125 g of Trolox in 1 ml of DMSO.

***Quencher solution (10 mM sodium ascorbate and 5 mM Trolox in 1xPBS)***

Prepare 10 ml of the quencher solution by mixing 0.1 ml of 1 M sodium ascorbate and 0.1 ml of 500 mM Trolox with 9.8 ml 1xPBS at room temperature.

***Regular-SDS RIPA lysis buffer (150 mM NaCl, 1 mM EDTA, pH 8.0, 50 mM Tris-HCl, pH 8.0, 1% NP40, 0.5% sodium deoxycholate, <u>0.1% SDS</u>)***

To make 100 ml, mix 3 ml of 5 M NaCl, 0.2 ml of 0.5 M EDTA, pH 8.0, 5 ml of 1 M Tris-HCl, pH 8.0, 1 ml of NP-40 (Igepal CA-630), 5 ml of 10% sodium deoxycholate, 1 ml of 10% SDS in 80.8 ml of nuclease-free water. Store at 4 °C.

***High-SDS RIPA lysis buffer (150 mM NaCl, 1 mM EDTA, pH 8.0, 50 mM Tris-HCl, pH 8.0, 1% NP40, 0.5% sodium deoxycholate, <u>0.5% SDS</u>)***

To make 100 ml, mix 3 ml of 5 M NaCl, 0.2 ml of 0.5 M EDTA, pH 8.0, 5 ml of 1 M Tris-HCl, pH 8.0, 1 ml of NP-40 (Igepal CA-630), 5 ml of 10% sodium deoxycholate, 5 ml of 10% SDS, and 80.8 ml of nuclease-free water. Store at 4 °C.

***B&W buffer (5 mM Tris-HCl, pH 7.5, 5 mM EDTA, 1 M NaCl, 0.1% Tween-20)***

To make 10 ml, mix 50 µl of 1 M Tris-HCl, pH 7.5, 100 µl of 0.5 M EDTA, pH 8.0, 2 ml of 5 M NaCl and 10 µl of 100% Tween-20, and 7.84 ml of nuclease-free water.

***Protease digestion buffer (300 mM NaCl, 150 mM Tris-HCl, pH 7.5, 3 mM EDTA, pH 8.0, 3% SDS)***

To make 10 ml, mix 600 µl of 5 M NaCl, 1.5 ml of 1 M Tris-HCl, pH 7.5, 60 µl of 0.5 M EDTA, pH 8.0, 3 ml of 10% SDS, and 4.84 ml of nuclease-free water. Store at room temperature. If precipitate is observed, briefly incubate at 37°C.

## B. HyPro-FISH

***Day 1 - Seeding the cells***

1. Seed ~1-1.5×$10^5$ HeLa cells in 1 ml of DMEM, 10% FBS and 1xPen-Strep per well of a 12-well plate with a sterile 18 mm round coverslip at the bottom.

***Day 2 - Fixation and permeabilization***

1. Wash the coverslips once with 1×PBS. Use sharp-tip forceps to handle the coverlips and keep the cell-containing side facing up throughout the entire protocol, except for the overnight hybridization and mounting steps (i.e. steps 9 and 24 described below).
2. Fix with freshly prepared 0.5 mg/ml DSP solution (~1 ml per coverslip) for 30-45 min at room temperature.
3. Wash 3 times with ~1 ml of 20 mM Tris, pH 7.5 in 1xPBS, 5 min each wash.
4. Permeabilize the cells with ~1 ml of 70% ethanol for 1 hour at room temperature or overnight at 4°C. (**Fixed cells can be kept in 70% ethanol for up to a week at 4°C**).

***Day 3 - Hybridization***

5. Rinse the coverslips in 2xSSC, 10% formamide (~1 ml) for 1-2 min.
6. Dilute DIG-labelled oligonucleotide probe set in the hybridization buffer (2xSSC, 10% formamide, 10% dextran sulfate) to the final concentration of 5 nM (45S), 25 nM (NEAT1 and the non-repetitive PNCTR probe set) or 50 nM (probe against the (UC)n repeats).
7. Spread a layer of parafilm on a flat surface (e.g. a plastic or glass plate) keeping the clean side up.
8. Spot 20 µl of the probe-containing hybridization mixture somewhere in the middle.
9. Pick up a coverslip with the forceps and blot any excess of the rinsing solution with a dry kimwipe. Do not touch the cell-containing side of the coverslip and do not allow this side to get dry. Place the coverslip on the top of the 20-µl drop with the cells facing down. Avoid trapping air bubbles between the coverslip and the hybridization mixture.
10. Cover with another sheet of parafilm, seal the edges, wrap the entire bundle (i.e. the base plate and the coverslip in the parafilm pocket) loosely in foil, and incubate in a

humidified chamber (we use a zip-lock bag with a few sheets of paper towel soaked with 2xSSC) at 37°C overnight.

*Day 4 - Proximity biotinylation*

11. Wash the coverslips with ~1 ml of 50% formamide/ 2xSSC at 37°C for 30 min.
12. Wash the fixed cells with ~1 ml of 1xSSC at room temperature for 15 min.
13. Block with 0.8% BSA in 4xSSC for 30 min at room temperature.
14. Incubate with HyPro protein diluted to 2.7 ng/ml in 4xSSC, 0.8% BSA, 0.1 units/µl RNasin (we use murine RNase inhibitor from NEB; cat# M3014) at room temperature for 1 hour in a humidified chamber.
15. Wash at room temperature once with 4xSSC, once with 4xSSC, 0.1% Triton X-100 and once with 4xSSC, 10 min each wash.
16. Incubate the coverslips in 400 µl 1xPBS for 5 min at room temperature. HyPro infusion control samples are incubated at this point in 400 µl 1xPBS supplemented with 5.4 ng/ml HyPro protein.
17. Add an equal volume (400 µl) of 1xPBS containing 1 mM biotin-phenol and 0.2 mM $H_2O_2$ and incubate for 1 min at room temperature. (**This means that labeling occurs in the presence of 0.5 mM biotin phenol and 0.1 mM $H_2O_2$ and the HyPro infusion control additionally contains 2.7 ng/ml HyPro protein**).
18. Quench the reaction with three changes of freshly prepared Quencher solution (10 mM sodium ascorbate and 5 mM Trolox in 1xPBS), ~1 min each wash.
19. Rinse with 4xSSC for 1-2min.
20. Incubate with Alexa Fluor 647-labeled streptavidin (1:200) in 4xSSC, 0.8% BSA, 0.1 units/µl RNasin in a humidified chamber at room temperature for 1 hour.
21. Wash at room temperature once with 4xSSC, once with 4xSSC, 0.1% Triton X-100 and once with 4xSSC, 10 min each wash.
22. Rinse with 1xPBS.
23. Counterstain with DAPI (0.5 µg/ml) in 1xPBS for 3 min and wash briefly with 1× PBS.
24. Mount coverslips on a clean glass slide with ProLong Gold Antifade reagent and cure overnight at room temperature in the dark.
25. Image using an epifluorescence microscope. We use a Zeiss Axio Observer 7 system equipped with an α Plan-Apochromat 100x/1.46 Oil DIC M27 objective and a Hamamatsu ORCA-Flash4.0 V3 Digital CMOS camera, and controlled by Zeiss ZEN 2.5 Blue software.

## C. HyPro-labeling of proteins

*Day 1 - Seeding the cells*

1. Seed ~$0.6x10^6$ HeLa cells per 10-cm dish in 12 ml of DMEM, 10% FBS and 1xPen-Strep. You will need a ~90% confluent plate the next day, so adjust the number of cells and the exact time of plating, if necessary.

*Day 2 - Fixation, permeabilization and hybridization*

2. Wash the cells once with ~5 ml of 1xPBS.
3. Fix with freshly prepared 0.5 mg/ml DSP solution (~5 ml) for 30-45 min at room temperature.
4. Wash 3 times with 20 mM Tris, pH 7.5 in 1xPBS (~5 ml), 5 min each wash.

5. Permeabilize the cells with 70% ethanol (~5 ml) for 1 hour at room temperature or overnight at 4°C.
6. Rinse with 2xSSC, 10% formamide (~5 ml) for 1-2 min.
7. Dilute DIG-labelled oligonucleotide probe mixture in the hybridization buffer (2xSSC, 10% formamide, 10% dextran sulfate) to 5 nM (45S), 25 nM (NEAT1 and the non-repetitive PNCTR probe set) or 50 nM ((UC)n-specific probe).
8. Add 6 ml of diluted probe mixture per dish. Seal the plate with parafilm and leave in a humidified chamber at 37°C overnight (we soak a few sheets of paper towel with 2xSSC and place them inside a zip-lock bag along with the 10 cm dish).

***Day 3 - Proximity biotinylation***

9. Wash with ~5 ml of 2xSSC, 10% formamide, for 30 min at 37°C.
10. Wash with ~5 ml of 1xSSC for 15 min at room temperature.
11. Block with ~5 ml of 4xSSC, 0.8% BSA, 100 units/ml RNasin for 30 min at room temperature.
12. Incubate with 5 ml of HyPro protein diluted to 2.7 ng/ml in 4xSSC, 0.8% BSA, 100 units/ml RNasin at room temperature for 1 hour in a humidified chamber.
13. Wash at room temperature once with 4xSSC, once with 4xSSC, 0.1% Triton X-100 and once with 4xSSC, 10 min each wash.
14. Incubate the cells in 4 ml of 1×PBS (experiment) or 1×PBS with 5.4 ng/ml HyPro protein (HyPro infusion control) for 5 min at room temperature.
15. Add an equal volume (4 ml) of 1xPBS containing 1 mM biotin-phenol and 0.2 mM $H_2O_2$ and incubate for 1 min at room temperature. (**This means that the labeling reaction occurs in the presence of 0.5 mM biotin phenol and 0.1 mM $H_2O_2$ and that the HyPro infusion control additionally contains 2.7 ng/ml HyPro protein**).
16. Quench the cells with three changes of freshly prepared Quencher solution.
17. Aspirate the solution and lyse the cells with 600 µl of high-SDS RIPA lysis buffer supplemented with 1× cOmplete EDTA-free protease inhibitor, 1 mM PMSF, 10 mM sodium ascorbate, 5 mM Trolox, 50 mM DTT and 100 units/ml RNasin. Swirl to spread the lysis buffer over the entire plate and incubate on ice for 5-10 min.
18. Scrape the lysed material off the plate and incubate for another 10 min on ice.
19. Split the lysate into ≤300-µl aliquots in 1.5-ml microfuge tubes and sonicate using Bioruptor set on "high", 30 sec ON / 30 sec OFF for 5-10 cycles. (**These settings may need to be adjusted depending on the cell line used**).
20. Incubate at 37°C for 30 min to reverse the DSP crosslinks.
21. Spin at 15,000xg for 10 min at 4°C. Lysates can be stored at -80°C at this point.
22. OPTIONAL: set aside 10-20% of each lysate to analyze biotinylated proteins by immunoblotting with streptavidin conjugates (step 31 below). (**Note that BCA protein quantification assay may not work properly in the presence of Trolox/ascorbate**).

***Days 4&5 - Isolation of biotinylated proteins and downstream analyses***

23. Wash 60 µl of streptavidin magnetic beads twice with regular-SDS RIPA. This amount of beads should be sufficient for SDS-PAGE analysis and running 3 technical replicates of label-free mass-spec.
24. Resuspend the beads in 3 ml regular-SDS RIPA and incubate with ~600 µl of lysates (0.5% SDS) at room temperature for 2 h with rotation (or overnight at 4°C).
25. Pellet the beads using magnetic rack and remove the supernatant (you can keep it in case troubleshooting is needed).

26. Wash beads twice with regular-SDS RIPA; once with 1 M KCl; once with 0.1 M $Na_2CO_3$; once with freshly prepared 2 M urea in 10 mM Tris-HCl, pH 8.0; and twice with regular-SDS RIPA to remove unspecific bound proteins. Analyze the samples by SDS-PAGE/immunoblotting (steps 27-31) or/and mass-spectrometry (steps 32-38).

***SDS-PAGE / immunoblotting***

27. Set aside ~1/6th of protein-loaded beads and incubate this aliquot with 15 µl regular-SDS RIPA supplemented with 1× cOmplete EDTA-free protease inhibitor, 1 mM PMSF, 5 mM biotin and 50 mM DTT for 20 min at 37°C with gentle agitation.
28. Add 15 µl of 4xLDS sample buffer supplemented with 50 mM DTT and incubate at 70°C for 10 min.
29. Vortex the beads briefly, cool the samples on ice and briefly spin down the samples to collect the condensation. Place the samples on magnetic rack to pellet the beads and analyze the eluate by SDS-PAGE / immunoblotting.
30. For abundant RNA baits, SDS-PAGE gel may be fixed and stained with SYPRO Ruby (Thermo Fisher Scientific), as recommended. Visualize the bands using a Typhoon 9210 or another scanner with appropriate excitation/emission filters.
31. For immunoblotting, electro-transfer SDS-PAGE-separated proteins to a nitrocellulose membrane using your favorite method (we use a Trans-blot system from Bio-Rad). Block the membrane with 3% BSA in 1xTBS, 0.1% Tween-20 for 30-60 min at room temperature or overnight at 4°C (the latter tends to produce cleaner backgrounds). Incubate with streptavidin-HRP (1:20,000 in 3% BSA in 1xTBS, 0.1% Tween-20) for 45-60 min at room temperature, wash four times with 1xTBS, 0.1% Tween-20 for 5 min at room temperature, and visualize the bands by ECL. Note that we often use immunoblotting to analyze HyPro-labeled lysates prior to capturing biotinylated proteins on magnetic beads (see step 22 above).

***Mass-spectrometry***

32. Wash the remaining beads (i.e. ~5/6th of the total volume) three times with 50 mM ammonium bicarbonate, pH 8.0 (AmBic).
33. Resuspend in 45 µl of 50 mM AmBic containing 1.5 µg of Trypsin/Lys-C mix (Promega). Incubate overnight at 37°C, with rotation.
34. On the next day, add an additional 0.75 µg Trypsin/Lys-C mix (Promega) in 15 µl 50 mM AmBic and incubate for another 2-3 h in a thermomixer set at 37°C.
35. Collect the beads and transfer the supernatant to a fresh tube.
36. Wash the beads twice with 45 µl of LC-MS grade water (90 µl in total) and combine the washes with the ~60 µl of supernatant collected at the pervious step.
37. Remove any particles by centrifuging at 14,000-16,000xg for 10 min.
38. Analyze the samples by label-free mass spectrometry.

## D. HyPro-labeling of RNAs

***Day 1 - Seeding the cells***

1. Seed ~$0.6 \times 10^6$ HeLa cells per 10-cm dish in 12 ml of DMEM, 10% FBS and 1xPen Strep. You will need a ~90% confluent plate the next day, so adjust the number of cells and the exact time of plating, if needed.

***Day 2 - Fixation, permeabilization and hybridization***

2. Wash the cells once with ~5 ml of 1xPBS.
3. Fix with freshly prepared 0.5 mg/ml DSP solution (~5 ml) for 30-45 min at room temperature.
4. Wash 3 times with 20 mM Tris, pH 7.5 in 1xPBS (~5 ml), 5 min each wash.
5. Permeabilize the cells with 70% ethanol (~5 ml) for 1 hour at room temperature or overnight at 4°C.
6. Rinse the fixed cells with 2xSSC, 10% formamide (~5 ml) for 1-2 min.
7. Dilute DIG-labelled oligonucleotide probe mixture in the hybridization buffer (2xSSC, 10% formamide, 10% dextran sulfate) to 5 nM (45S), 25 nM (NEAT1 and the non-repetitive PNCTR probe set) or 50 nM ((UC)n-specific probe).
8. Add 6 ml of diluted probe mixture per dish. Seal the plate with parafilm and leave in a humidified chamber at 37°C overnight. Soak a few sheets of paper towel with 2xSSC and place inside a zip-lock bag along with the 10 cm dish to create a humidified environment.

***Day 3 - Proximity biotinylation***

9. Wash with ~5 ml of 2xSSC, 10% formamide, for 30 min at 37°C.
10. Wash with ~5 ml of 1xSSC for 15 min at room temperature.
11. Block with ~5 ml of 4xSSC, 0.8% BSA, 100 units/ml RNasin for 30 min at room temperature.
12. Incubate with 5 ml of HyPro protein diluted to 2.7 ng/ml in 4xSSC, 0.8% BSA, 100 units/ml RNasin at room temperature for 1 hour in a humidified chamber.
13. Wash at room temperature once with 4xSSC, once with 4xSSC, 0.1% Triton X-100 and once with 4xSSC, 10 min each wash.
14. Incubate the cells in 4 ml of 1xPBS (experiment) or 1xPBS with 5.4 ng/ml HyPro protein ("HyPro infusion control") for 5 min at room temperature.
15. Add an equal volume (4 ml) of 1xPBS containing 1 mM biotin-phenol and 0.2 mM $H_2O_2$ and incubate for 1 min at room temperature. (**The labeling reaction occurs in the presence of 0.5 mM biotin phenol and 0.1 mM $H_2O_2$ and that the HyPro infusion control additionally contains 2.7 ng/ml HyPro protein**).
16. Quench the cells with three changes of freshly prepared Quencher solution (10 mM sodium ascorbate and 5 mM Trolox in 1xPBS).
17. Aspirate the solution and lyse the cells with 600 µl of high-SDS RIPA lysis buffer supplemented with 1x cOmplete EDTA-free protease inhibitor, 1 mM PMSF, 10 mM sodium ascorbate, 5 mM Trolox, 50 mM DTT, and 100 units/ml RNasin. Swirl to spread the lysis buffer over the entire plate and incubate on ice for 5-10 min.
18. Scrape the lysed material off the plate and incubate for another 10 min on ice.
19. Split the lysate into ≤300-µl aliquots in 1.5-ml microfuge tubes and sonicate using Bioruptor set on "high", 30sec ON/30 sec OFF for 5-10 cycles. (**These settings may need to be adjusted depending on the cell line used**).
20. Incubate at 37°C for 30 min to reverse the DSP crosslinks.
21. Add 20 µl of proteinase K (20 mg/ml) and incubate at 50°C for 1 hour. The solution should become clear at the end of this step.
22. Mix the lysate with 3 volumes of Trizol LS reagent (e.g. 0.75 ml of TRIzol LS per 0.25 ml of the lysate).
23. Incubate for 5 minutes at room temperature.
24. Add 0.2 ml of chloroform per each 0.75 ml of TRIzol LS used at the previous step, shake vigorously for 15 sec and incubate for 2–3 minutes at room temperature.
25. Centrifuge the sample for 15 minutes at 12,000×g at 4°C to separate the phases.

26. Transfer the aqueous phase to a new tube and extract total RNA using a Purelink RNA miniprep kit (Thermo Fisher) with on-column DNase digestion.
27. Elute RNA in 100 µl of RNase-free water. Measure RNA concentration e.g. using a NanoDrop spectrophotometer. In our hands, a 10-cm dish of HeLa cells yields ~30-50 µg of total RNA.
28. Set aside 5% of the total RNA as the input fraction for RT-qPCR analysis. Use the remaining sample to isolate biotinylated RNA species.

***Day 4 - Isolation of biotinylated RNAs and downstream analyses***

29. We use 20 µl of MyOne streptavidin C1 magnetic beads per 50 µg of total RNA. Wash the required volume of beads three times with the B&W buffer; once in 0.1 M NaOH, 0.05 M NaCl; once in 0.1 M NaOH, 0.05 M NaCl, 0.1% Tween-20; once in 0.1 M NaCl; and once in 0.1 M NaCl, 10 mM Tris-HCl, pH 7.5, 1 mM EDTA, 0.2% Tween-20. Resuspend the beads in 150 µl of 0.1 M NaCl, 10 mM Tris-HCl, pH 7.5, 1 mM EDTA, 0.2% Tween-20, 0.2 units/µl RNasin.
30. Mix the beads with total RNA topped up with nuclease-free water to 150 µl. (Final volume of the RNA-bead slurry is ~300 µl).
31. Incubate at 4°C for 2 hours, with rotation.
32. Collect the beads using a magnetic rack, remove the supernatant (you can keep it in case troubleshooting is needed).
33. Wash the beads 3 times with the B&W buffer and resuspend in 63 µl nuclease-free water, 33µl of 3x protease digestion buffer (300 mM NaCl, 150 mM Tris-HCl, pH 7.5, 3 mM EDTA, 3% SDS), and 4 µl of proteinase K (20 mg/ml).
34. Incubate at 50°C for 45 min, with agitation.
35. Add 3 volumes of Trizol LS, mix, incubate for 5 min, add 0.2 ml chloroform per each 0.75 ml of Trizol LS used, mix vigorously, and incubate at room temperature for 2-3 min.
36. Centrifuge the sample for 15 minutes at 12,000×g at 4°C.
37. Purify biotinylated RNA from the aqueous phase using an RNA clean and concentrator kit (Zymo Research), as recommended. For consistency, clean up the input RNA aliquot set aside at the end of the previous section (*Day 3*) using the same RNA clean and concentrator kit routine.
38. Elute RNAs in 15 µl of nuclease-free water.
39. Set aside a few microliters to measure RNA concentration and optionally check pull-down specificity by RT-qPCR with primers against the RNA bait and a housekeeping control (e.g. GAPDH). Since the amount of biotinylated RNA recovered from streptavidin beads is typically just a few nanograms, measure the concentration using a Qubit with an RNA HS Assay kit (Thermo Fisher Scientific). We like the RNA spike-in method by Li et al. (2015) BMC Mol. Biol. 16:9.
40. Store the rest at -80°C for subsequent RNA-seq analyses.
